# Explaining the Imperfection of the Molecular Clock of Hominid Mitochondria

**DOI:** 10.1371/journal.pone.0008260

**Published:** 2009-12-29

**Authors:** Eva-Liis Loogväli, Toomas Kivisild, Tõnu Margus, Richard Villems

**Affiliations:** 1 Estonian Biocentre, Tartu, Estonia; 2 Department of Evolutionary Biology, University of Tartu, Tartu, Estonia; 3 Leverhulme Centre of Human Evolutionary Studies, University of Cambridge, Cambridge, United Kingdom; 4 Department of Bioinformatics, University of Tartu, Tartu, Estonia; University of Utah, United States of America

## Abstract

The molecular clock of mitochondrial DNA has been extensively used to date various genetic events. However, its substitution rate among humans appears to be higher than rates inferred from human-chimpanzee comparisons, limiting the potential of interspecies clock calibrations for intraspecific dating. It is not well understood how and why the substitution rate accelerates. We have analyzed a phylogenetic tree of 3057 publicly available human mitochondrial DNA coding region sequences for changes in the ratios of mutations belonging to different functional classes. The proportion of non-synonymous and RNA genes substitutions has reduced over hundreds of thousands of years. The highest mutation ratios corresponding to fast acceleration in the apparent substitution rate of the coding sequence have occurred after the end of the Last Ice Age. We recalibrate the molecular clock of human mtDNA as 7990 years per synonymous mutation over the mitochondrial genome. However, the distribution of substitutions at synonymous sites in human data significantly departs from a model assuming a single rate parameter and implies at least 3 different subclasses of sites. Neutral model with 3 synonymous substitution rates can explain most, if not all, of the apparent molecular clock difference between the intra- and interspecies levels. Our findings imply the sluggishness of purifying selection in removing the slightly deleterious mutations from the human as well as the Neandertal and chimpanzee populations. However, for humans, the weakness of purifying selection has been further exacerbated by the population expansions associated with the out-of Africa migration and the end of the Last Ice Age.

## Introduction

The idea of genes accumulating new mutations in a clock-like manner provides a versatile tool for dating genetic events [1], [2] even when the clock is stochastic and its rate variable [3]. Time estimates are essential in phylogeographic interpretations of population subdivisions, expansions, migrations and colonization of new areas [4]. Phylogeographic studies of human mitochondrial DNA (mtDNA), especially those based on complete mitochondrial genomes, have yielded many results of general interest concerning the origins of our species and population movements that are behind the genetic patterns seen today [5], [6], [7]. The coalescent time or the time of the first expansion of a clade is often measured by the average amount of mutations that have accumulated since the emergence of a particular node in the tree [8]. Until recently, a linear molecular clock for the human mtDNA has been assumed and applied for dating the nodes of the tree [9]. On the other hand, time-dependence of the rate of accumulation of new mutations has been proposed based on several lines of evidence [10], [11], [12], [13], [14], [15], [16]. Notably, the phrase “accumulation of new mutations” can refer to different levels of genetic variation: it can be related to DNA replication errors left unrepaired in meiosis (observed in pedigree studies), however, in population studies it refers to the level of polymorphism and in interspecies comparisons to the number of fixed differences. Time-dependence of mtDNA clock derives from the apparent substitution rate differences observed between the pedigree, intra- and interspecies levels.

It is claimed that the mutation rate at the pedigree level, just a few generations of time depth, is an order of magnitude higher if compared to the substitution rates found in evolutionary analyses [17], [18], but see [9]. In addition, for various species the relative rate of non-synonymous substitutions (K_a_/K_s_) is found to be higher among populations than in interspecies comparisons [19], [20], [21]. Furthermore, non-synonymous substitutions were shown to be more frequent in terminal branches of the human mtDNA tree [22], [23], [24] and in younger clades compared to older clades [25]. There appears to be a continuous change in the mtDNA substitution rate with the slowest rate in between humans and apes, intermediate rate among human populations and the highest rate in pedigrees [13], [26].

The action of purifying selection against non-synonymous mutations at the level of mitochondrial transmission has been observed directly in experiments with mice expressing proofreading deficient mtDNA polymerase [27]. Indeed, the existence of slightly deleterious substitutions, which contribute to population polymorphism but not to interspecies differences, has been a favored explanation [14], [15], [20], [21], [28]. In addition, demographic history of a serial of bottlenecks prior to the Holocene has been proposed to explain the difference between pedigree and population-level substitution rates [14]. Undetected saturation at mutational hotspots could also be responsible for the apparent rate differences, particularly in between population and interspecies levels [14], [25]. Whereas, studies claiming that saturation is not causing the apparent synchronous rate change at the interspecies levels of both, primates and birds [10], [29], have met considerable criticism on the grounds of methodology and data quality [9], [30], [31].

It has been argued that the excessive accumulation of non-synonymous mutations has continued gradually since the out-of-Africa migration of humans [32] and that the gradual rise in K_a_/K_s_ can be traced back even over millions of years [29]. Others have proposed a more recent, possibly sudden shift related only to the external branches of the mtDNA tree [22], [24], [33] or that the excess of non-synonymous mutations concerns only clades that are less than 10,000 years old [9]. Recently, new evidence has been accumulating describing the mode and extent of the apparent rate change over time [34]. Two recent studies are based on the fact that the possible rate change must cause the choice of calibration point to have an effect on the resulting substitution rate estimate [12], [14]. These authors have chosen various archaeological dates of demographic expansions and measured the genetic diversity that has accumulated in the phylogenetic clades associated with these expansions to calibrate the mtDNA clock. Endicott and Ho (2008) [12] used 3 dates to achieve a single population level rate calibration for human mtDNA in a Bayesian analysis. Henn et al (2009) [14] used 14 calibration points for the first hypervariable region (HVRI) and 11 calibration points for the coding region median-joining networks of mtDNA. Both studies found that intra-human substitution rates were higher compared to the rate calibrated from the human-chimpanzee divergence point. The use of multiple calibration points enabled Henn et al (2009) [14] to observe a sudden shift in substitution rate at about 15 thousand years ago (kya) in both, HVRI and coding regions.

However, the approach chosen by these studies [12], [14], relies on archaeological dates and associations between demographic events and specific clades, some of which, like the first settlement of Australasia, are controversial. In addition, provided that the diversification of haplogroups H1 and H3 was associated with the re-settlement of Europe after the Last Glacial Maximum (LGM) and the Younger Dryas, the expansion time of 18 ky, applied by both studies, could be an overestimation. This kind of mistake would make the substitution rate appear slower and exaggerates the difference with the pedigree rate. In an alternative approach, one can avoid archaeological dates as calibration points and instead measure the time using the amount of neutral variation that is expected to accumulate in a clock-like manner. Such an approach was taken in a recent study [16]. However, in this study the haplogroup age estimates, both absolute and relative, were at odds with the general view on the timeline of settlement of Eurasia by modern humans [34]; for instance, haplogroups U and H both showed divergence times of about 75 kya, which is difficult to envision [15], [35]. While our manuscript was under review, a thorough study on mtDNA mutation rates was published, which also relies on genetic rather than archaeological dates [15]. The authors assumed a monotonic decline in the proportion of non-neutral mutations and fitted the apparent total substitution rate with a simple growth distribution. The resulting distribution was applied to correct for the haplogroup age estimates derived from the total variation observed in the mitochondrial genome [15].

We studied the apparent substitution rate change of human mtDNA as a continuous process without relying on associations of particular clades and demographic events. Interspecies time-scale was covered by mtDNA comparisons between human and chimpanzee, human and Neandertal and between two chimp lineages. Protein and RNA genes were analyzed separately to reveal their relative share in the apparent rate change. The obtained results were further tested for the effects of site-specific positive selection and sequencing errors. As the molecular clock of human mtDNA was expected to be non-linear, we applied only synonymous substitutions to measure the clade ages and interspecies divergence times. However, the amount of synonymous variation that has accumulated in between humans and chimpanzees has been difficult to accommodate with variation observed within our species. The distance between humans and chimps is significantly less than predicted from the rate of substitution observed within humans and correction for multiple hits under uniform rate model is not capable of explaining the difference. Therefore, we also studied mutational saturation under a model of rate variation between synonymous sites. The possible reasons and implications of the non-linearity of the human mtDNA clock are discussed.

## Results

The Mitomap tree of 3057 human mitochondrial DNA coding region sequences (Table S1, Figure S1) was analyzed for time dependent changes in the relative frequencies of substitutions. Time depth was measured by the genetic distance in synonymous substitutions. Relative substitution rates of non-synonymous and RNA gene substitutions were determined in respect of synonymous substitutions for 186 clades. Altogether, 8140 nucleotide substitutions were counted on the tree, 4535 of which (55.7%) were synonymous (S), 2135 (26.2%) were non-synonymous (N) and 1470 (18.1%) involved RNA and intergenic (RI or RNA) mutations. The overall average ratio of non-synonymous and synonymous mutations was 0.471 (N/S) and that of RNA and synonymous was 0.324 (RI/S). If the number sites that enabled such mutations was taken into account, this would correspond to the relative rates of 0.274 for K_a_/K_s_ and 0.329 for K_ri_/K_s_.

**Figure 1:**
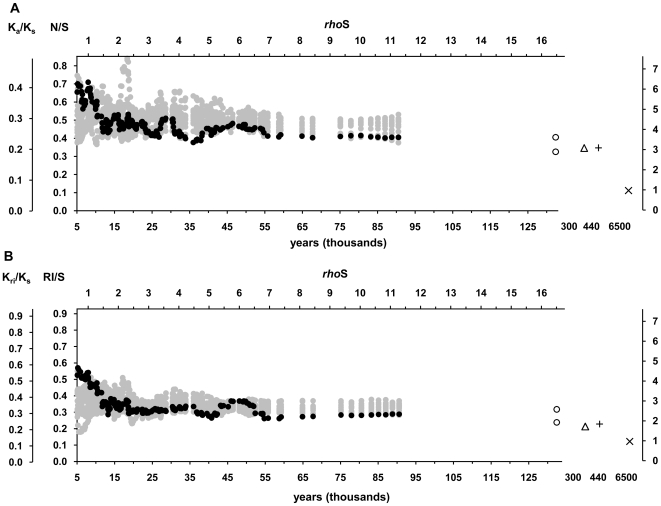
Temporal changes in the fractions of non-synonymous (A) and RNA plus intergenic mutations (B). N/S – average ratio of non-synonymous to synonymous mutations of the clades belonging to the same time-window. K_a_/K_s_ – relative substitution rate of non-synonymous mutations per non-synonymous site in respect of the rate of synonymous mutations per such a site. RI/S and K_ri_/K_s_ give the relative mutation rates of RNA genes plus intergenic regions. Black dots represent the estimates based on the empirical dataset. Grey dots show the estimates from the reference dataset with a uniform distribution of mutations. Open circles show the values for the 4 oldest clades. The lower open circle shows the substitution rate without counting the mutations from the youngest branches, those that carried only 1 or 2 individuals. Open triangle shows the substitution rate between two chimpanzee sequences (X93335 [58] and EU095335 [56]). The plus sign and cross represent data points for the comparisons of human(rCRS)-Neandertal [60] and human(rCRS) [40]-chimpanzee [56], respectively. The upper axis (*rhoS*) gives the ages as average amount of accumulated synonymous substitutions. The lower axis shows the ages in calendar years, with 1 *rhoS* corresponding to 7990 years. The right axis gives relative changes in respect of the substitution rate inferred from the human-chimpanzee comparison. See the details of the sliding window analysis and estimates for the Neandertal and chimpanzee sequences in the text and *Methods*.

Mutation ratios of the 186 clades were sorted by coalescence ages of the clades and averaged over a “time-window” of 15 clades with similar coalescence ages in a sliding window analysis. Throughout this study, the ages have been measured by the newly calibrated substitution rate, which is 3×10^−8^ synonymous substitutions/site/year that corresponds to 7990 years per any synonymous substitution in the mitochondrial genome (or 8250 years per synonymous transition). Both ratios, for the non-synonymous (N/S) and RNA gene substitutions (RI/S), exhibited a trend of increase with diminishing clade ages (Figure 1A, B). Both had their maxima at the young end of the time scale and these values were about 1.7 times greater than the mutation ratios found in the oldest clades. The results presented in Table 1 and Figure 1 underestimate the differences because the high ratios of the younger branches also contributed to the ratios recorded in the older clades. Indeed, after the exclusion of mutations from the branches that carried only 1 or 2 individuals from the oldest clades, the rate differences extended to 2 and 2.3 fold for the non-synonymous and RNA gene substitutions, respectively. After the correction, the substitution ratios of the oldest clades were comparable with the ratios seen in the distance between human and Neandertal mtDNA or between two chimpanzees (Figure 1). At the same time these values were still 2–3 times higher than the rates derived from a human-chimpanzee comparison. The latter ratio was considered to be stable because negative selection would have wiped out all deleterious substitutions since the split of the lineages of the two species. Notably, about a half of the observed rise from the stable rate in RNA gene and a third of the rise in non-synonymous substitutions took place only after the end of the Last Ice Age, 11.7 ky ago. Both empirical curves significantly differed from a simulated dataset with uniform distribution of substitutions over the entire tree (Figure 1, Table 1). The empirical mutation ratios exceeded the average simulated values in the younger end of the time-scale but were lower in the older end. This pattern is compatible with a scenario of purifying selection gradually removing the slightly deleterious non-synonymous and RNA mutations from the population.

**Table 1:** The extent and significance of temporal changes in the mutation ratios.

window#^a^	age (*rhoS*)^b^	N/S		RI/S		all subst/S	
		ratio^a^	p^c^	ratio^a^	p^c^	ratio^d^	e
1–10	0.64–0.88	0.639	***	0.525	***	2.16	1.75
11–20	0.90–1.09	0.652	***	0.496	***	2.15	1.74
21–30	1.10–1.44	0.570	*	0.450	***	2.02	1.64
31–40	1.47–1.65	0.478	**	0.348		1.83	1.48
41–50	1.66–1.88	0.477	*	0.344		1.82	1.47
51–60	1.88–2.04	0.503		0.338	**	1.84	1.49
61–70	2.06–2.30	0.474	*	0.346	**	1.82	1.47
71–80	2.30–2.43	0.502		0.320	**	1.82	1.47
81–90	2.45–2.79	0.475	**	0.308	***	1.78	1.44
91–100	2.79–3.00	0.461	**	0.304	***	1.77	1.43
101–110	3.00–3.41	0.426	***	0.311	***	1.74	1.41
111–120	3.42–3.84	0.493	***	0.317	***	1.81	1.46
121–130	3.87–4.50	0.418	***	0.325	***	1.74	1.41
131–140	4.61–5.10	0.417	***	0.286	***	1.70	1.38
141–150	5.11–5.75	0.453	***	0.315		1.77	1.43
151–160	5.81–6.50	0.460	***	0.356	***	1.82	1.47
161–170	6.55–8.47	0.430	***	0.277	***	1.71	1.38
171–178	9.40–11.33	0.408	***	0.285	***	1.69	1.37
1–178	0.64–11.33	0.486	**	0.348		1.83	1.48

*** p<0.001.

** p<0.01.

* p<0.05.

N – non-synonymous.

S – synonymous.

RI – RNA and intergenic.

a data from the sliding window analysis was further averaged over 10 windows, only empirical data is shown.

b median *rhoS* values for the first and last windows in the group of 10.

c p-values for the Aspin-Welch Unequal-Variance t-test comparisons of the empirical and the reference data.

d ratio of total variation to synonymous substitutions.

e multiple of 1.236, the human-chimpanzee interspecies value for the ratio of all substitutions to synonymous substitutions.

**Figure 2:**
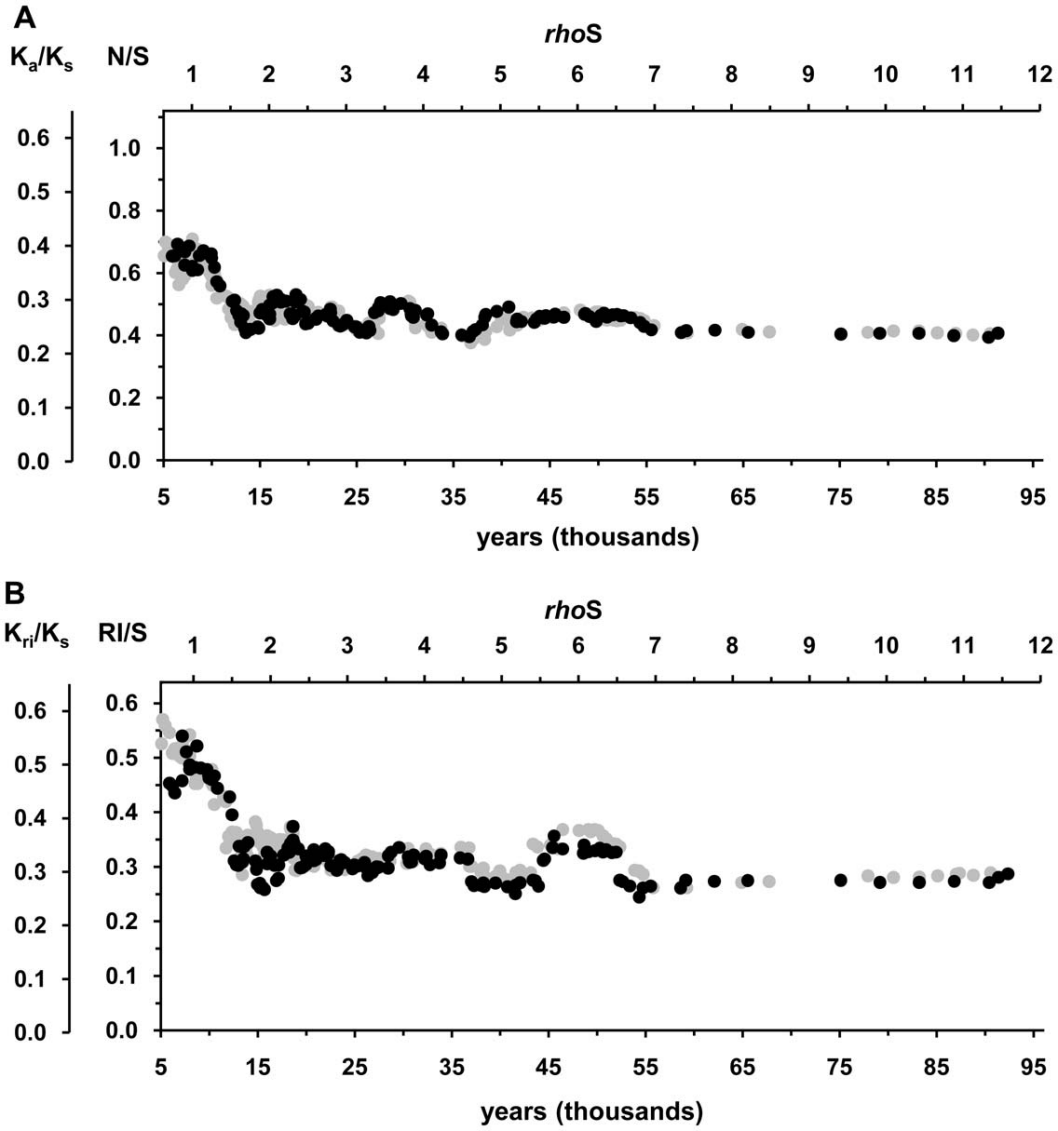
Temporal changes in the fractions of non-synonymous (A) and RNA plus intergenic mutations (B) after the removal of sub-trees defined by mutations favored by positive selection. These mutations were A5539G, A5592G, T7581C, G7521A, G1438A, T1452C, G3010A, T3027C and A12172G. Black dots represent the results after the removal of the mutations and grey dots show the results of the full dataset (Figure 1). The upper axis (*rhoS*) gives the ages as average amount of accumulated synonymous transitions. The lower axis shows the ages in calendar years, with 1 *rhoS* corresponding to 7990 years. The sliding window analysis was performed as with full dataset, except that 143 windows were analyzed. See the additional details in the text, Figure 1 legend and *Methods*.

Although there is no evidence of strong directional selection on human mtDNA [23], [25], [32], [36], it is possible that weak positive selection acts on many branches of mtDNA tree [25], [33]. Indeed, if positive selection favored many younger branches, it could result in a substitution pattern similar to that produced by incomplete purifying selection. To test whether the influence of positive selection could have caused the apparent rate acceleration, we excluded all sub-trees defined by mutations at evolutionarily conserved sites that have shown evidence of back-mutation to a more favorable state [25], [33]. This included mutations that restored Watson-Crick base pairing in 5 RNA stems: A5539G in tRNA^Trp^; A5592G in tRNA^Ala^; T7581C or G7521A in tRNA^Asp^; G1438A or T1452C in 12S rRNA; G3010A or T3027C in 16S rRNA gene. In addition, A12172G in tRNA^His^ gene that changed the nucleotide that was conserved in >90% of 31 mammalian species was removed. Altogether, 883 individual sequences were removed from the tree and the number of clades in the sliding window analysis reduced to 151. Nevertheless, the patterns of substitution ratios changed only marginally (Figure 2). We conclude that the overall effect of positive selection on the substitution pattern of mtDNA is weak.

Synonymous changes are assumed to be free of strong natural selection. Despite of that, the effect of apparent rate change has also been observed for synonymous mutations. Both undetected saturation and variation of substitution rates in between synonymous sites have been proposed as the main factors behind the discrepancy between the intra- and interspecies apparent substitution rates [9], [14], [25]. Rate variation among sites can be caused by basic molecular biases like strand asymmetry or by natural selection. We studied the rate variation of synonymous sites by analyzing the distribution of the number of synonymous sites by mutation hits they had received (Table 2). The number of mutation hits at each synonymous site was counted on the Mitomap tree of 3057 sequences. A table was created showing the number of sites and the respective number of mutation hits the sites had received (Table 2). We then fitted the summary distribution of several Poisson distributions, generated with variable number of different rates, with the observed values. Half of the sites were invariable in the dataset; however, it is unlikely that these synonymous sites were not free to vary, which was also indicated by the poor fit with the expected distribution when only the variable sites were considered (1 rate: p<10^−136^ (χ^2^ 697, df 17); 3 rates: p<10^−28^ (χ^2^ 180, df 17)). Fitting the expected distributions with the observed distribution for all sites, variable and invariable, revealed that the overall average rate of 1.08 synonymous substitutions per such a site did not describe the rate of evolution well (p<10^−189^, χ^2^ 949, df 18). We found that 71% of the sites had evolved more than twice slower (37%) than average (Table 3). 25% of the synonymous sites had a substitution rate nearly 2 times faster than average and 4% of the sites had evolved about 6 times faster compared to the average rate. The finding that just 3 discrete classes of substitution rates can explain rate variation at synonymous sites stands somewhat in contrast to earlier assumptions of a continuous gamma distribution of substitution rates over all coding sites [15], [37], [38].

**Table 2:** Distribution of synonymous sites by the number of received mutation hits.

hits*	sites
0	2126
1	1073
2	449
3	217
4	107
5	70
6	32
7	30
8	22
9	13
10	11
11	3
12	3
13	5
14	2
15	2
16	1
17	1
19	1
28	1
35	1

* The data was copied from the Mitomap tree with no correction for multiple hits at the sites implicated in reticulations.

**Table 3:** Distribution of synonymous sites according to 3 rates of substitution.

sites (%)	rate* (%^a^)
2967 (71)	0.40 (37)
1042 (25)	1.99 (184)
161 (4)	6.54 (604)
4170	1.03^b^

a the rate of 1.08 mutations per position (4512/4170) counted from the Mitomap tree of 3057 mtDNA coding sequences was taken as the average rate (100%) for all synonymous positions.

b weighed average of the 3 rates of substitution.

* the best fit between the empirical and the expected summary distribution of the 3 sets of sites with different average rates shows non-significant difference (p = 0.67, χ^2^ 14.9, df 18).

The observed human-chimpanzee distance of 860 synonymous transitions was used to test the applicability of the 3-rates model of synonymous sites for the correction of the genetic distance for multiple mutation hits. The high level of divergence, 21%, implied a substantial amount of multiple hits. Assuming equal probability for all transitions, the correction would stretch the distance to 1109 mutations (Table 4). This, however, would yield a divergence date of 4.6 (SD 0.18) million years (my), which is at odds with paleontological findings that place the divergence of human lineage at least at 6 my ago [39]. We took the commonly referred date of 6.5 my for the split of the two species' mtDNA lineages and calculated the expected number of observed transitions under the 3-rates model. The obtained distance, 849 synonymous transitions, was only insignificantly different from the empirical value of 860, showing that it is sufficient to assume only 3 different substitution rates for the correction (Table 4). Notably, the common calculation of corrected distances from the observed differences was problematic for several reasons. First of all, the sites with the highest rate were expected to be fully saturated. In fact, they were even more divergent than would be expected by chance alone: the proportion of transitional differences significantly exceeded 50% (Table 5). This pattern of inter-species “over-divergence” was clearly concordant with the intraspecies substitution rate and went up to 80% for the sites that had mutated more than 10 times in the human mtDNA tree. To exclude the possible effect of recent rapid growth of human populations, the reference sequence (rCRS [40]) that represents extant variation was replaced by the reconstructed root sequence of the human mtDNA tree. The resulting difference was still significant for the sites that had mutated at least 6 times in the human mtDNA tree (Table 5).

**Table 4:** Comparison of the 1 rate and 3-rates models of synonymous transitions using the expected number of observed differences with the assumption of 6.5 my for the date of the human-chimpanzee lineage split.

		no of sites^a^	corr dist (K)^b^	dist (p)^c^
observed		4170	1109 (0.266)	860* (0.206)
expected (1 rate)		4170	1576 (0.379)	1106* (0.265)
expected (3 rates)		2967	428 (0.144)	372 (0.125)
		1042	748 (0.718)	397 (0.381)
		161	380 (2.362)	80 (0.496)
sum(average)		4170	1556 (0.373)	849* (0.204)**

a number of synonymous sites included in each rate class.

b corrected distance in synonymous transitions (per site). Jukes and Cantor (1969) [61] model of DNA substitution was modified to account only for transitions.

c expected human(rCRS) [40]-chimpanzee [56] distance to be observed in synonymous transitions (per site).

* The expected number of synonymous transitions derived from the 3-rates model is insignificantly different from the observed data but with the 1-rate model it is significantly different, according to Poisson distribution of mutations.

** The expected standard deviation of the distance (p), 0.87, is large due to saturation at the sites with high substitution rate.

**Table 5:** Proportion of observed transitional differences at synonymous sites between a human and a chimpanzee [56] sequences according to the sites' substitution rate.

rate^b^	sites	rCRS^a^		root	
		trs	prop.	trs	prop.
0	2126	296	0.138	294	0.138
1	1073	215	0.190	205	0.191
2	449	114	0.252	112	0.249
3	217	84	0.392	84	0.387
4	107	44	0.430	48	0.449
5–7	132	69	0.508	66	0.500
6–8	84	51	0.583	49	0.583
7–9	65	45	0.692***	44	0.677[Table-fn nt124] **
8–10	46	35	0.761***	34	0.739***
>10	20	16	0.800[Table-fn nt124] **	14	0.700*

a the corrected Cambridge Reference Sequence [40] or the reconstructed root of the human mtDNA tree (as in Behar et al. (2008) [62]) was used for comparison.

b the number of hits the sites have received in the Mitomap tree.

* p<0.05 in comparison with a binomial distribution with a mean of 0.5.

** p<0.01.

** p<0.001.

trs – transitions, prop. – proportion of different nucleotides.

## Discussion

**Figure 3:**
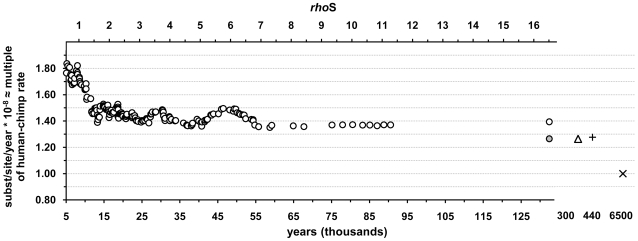
Changes in the overall substitution rate of the coding region. The rates shown are approximately the same as multiple of the rate derived from the human-chimpanzee comparison because this rate is close to 1 substitution/site/year. The last open circle shows the value for the 4 oldest clades. The grey circle shows the apparent substitution rate for the 4 oldest clades without counting the mutations from the youngest branches, those that carried only 1 or 2 individuals. Open triangle shows the substitution rate derived from the comparison of two chimpanzee sequences. The plus sign and cross represent data points for the comparisons of human-Neandertal and human-chimpanzee, respectively. The upper axis (*rhoS*) gives the ages as average amount of accumulated synonymous substitutions. The lower axis shows the ages in calendar years. See the details in Figure 1 legend and in the *Methods*.

The overall apparent substitution rate of mtDNA coding region shows no notable change for hundreds of thousands of years implying that the decrease of non-neutral mutations with time must be very slow (Figure 3). This observation is based on the human-Neandertal comparison and is supported by a comparison between two chimpanzees. We have dated the coalescence ages of these lineage splits according to our newly revised rate of synonymous transitions and by the application of the 3-rates model. This yielded a date of 440 (SD 138) ky ago for the divergence of the human and Neandertal mtDNA lineages. This date estimate makes it unlikely that the direct ancestors of Neandertals would have been around in Europe 800 ky ago, assuming that the human-neanderthal split occurred in Africa. However, the commonly referred divergence dates for the two species, (a) 400–600 kya, assuming the continuity between *H. heidelbergensis* and *H. neanderthalensis* in Europe [41] and (b) 250–300 kya, assuming their split from an intermediate species *H. helmei* in Africa [42], both lay within the error margins of our coalescence estimate. The latter hypothesis seems though more consistent with the mtDNA date estimate as it allows for a reasonable convergence time of DNA lineages within the ancestral species: if the species split occurred 250–300 kya then the additional 140–190 ky could be reserved for the coalescence of mtDNA lineages within the ancestral population, which would be comparable to the observed value in modern humans – about 190 ky.

**Figure 4:**
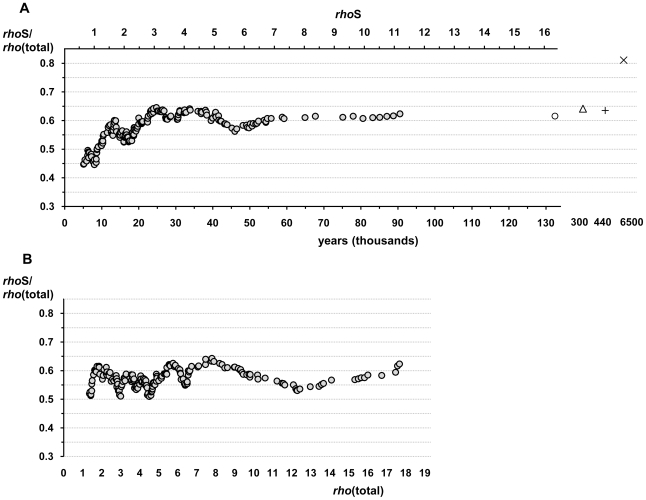
Changes in the fraction of synonymous substitutions. The dataset of coalescence ages was sorted according to the accumulated amount of synonymous variation (A) or total variation (B) and the sliding window analysis was performed as with the other mutation ratios (see *Methods*). Open circle shows the values for the 4 oldest clades. Open triangle shows the substitution rate derived from the comparison of two chimpanzee sequences. The plus sign and cross represent data points for the comparisons of human-Neandertal and human-chimpanzee, respectively. See the details in Figure 1 legend and in the *Methods*. Note that the time axis derived from total variation on panel B is in fact not linear, contrary to what is shown in panel A. The values on panel B have been stretched out along the younger end of the dataset because of the generally higher fraction of non-synonymous and RNA gene mutations there.

Our results imply that the change in the apparent substitution rate of mtDNA coding region involves fluctuations (Figure 3). In addition, the growth trend in the proportion of synonymous substitutions along with growing clade ages is wavy (Figure 4A). Furthermore, this growth trend is lost after sorting the data according to the accumulated total variation, which includes substitutions that are under selection (Figure 4B). The fluctuations in the apparent substitution rate imply interrelated influences of variable strength in natural selection and population size changes. Indeed, the earliest turning point in the apparent substitution rate coincides with the population expansion following the out-of Africa migration of modern humans, associated with the first diversification of the two mtDNA superclades M and N. Notably, a recently proposed correction for the molecular clock of mtDNA was based on a growth function derived from a dataset of proportions of synonymous substitutions that was sorted, like in Figure 4B, according to the accumulated total variation [15]. It is possible that the distortion of the growth trend is more evident in our dataset compared to theirs [15] because of the larger proportion of non-neutral variation in our data due to the exclusion of the non-coding control region. Detailed understanding of the behavior of mtDNA substitution rate would certainly increase our knowledge on the factors that have conditioned the development of existing genetic variation in human populations.

Our intrahuman data show systematically lower fraction of synonymous substitutions compared to the results of Soares et al (2009) [15]. For instance, the proportion of synonymous substitutions (0.62) found in the clades of about 140 ky makes up 75% of the respective proportion in the human-chimp distance (Figure 4A). The same comparison yields a fraction close to 90% in the dataset of Soares et al (2009). Unaccounted saturation cannot be the explanation for the discrepancy as the extent of saturation in the long branches of human mtDNA tree is very low [15]. The difference in the results of the two studies, however, has implications for the strength of purifying selection. According to Soares et al (2009) [15] the proportion of synonymous changes would be virtually stable by the time of the divergence of human and Neandertal mtDNA lineages, indicating only marginal role of purifying selection on mutations that have preserved from this time depth. In our dataset, however, the proportion of synonymous substitutions for that time point is only 78% of the respective human-chimp ratio. An indirect support for the weakness of purifying selection comes from the comparison of the two chimp sequences that diverged about 300 ky ago, which show a similar fraction of synonymous substitutions to the human-Neandertal pair. Notably, the estimates for the fraction of synonymous substitutions for the human-chimp distance were similar in our study and Soares et al. (2009): the proportion was 0.81, based on 3 rate classes for the synonymous sites and Jukes-Cantor correction for the distance in non-synonymous and RNA genes substitutions, and it was 0.79, assuming 1.55 times higher substitution rate for the coding region compared to the control region, as determined by Soares et al. (2009) [15].

Our substitution rate of 7990 years per synonymous mutation is slightly slower than the average result of Soares et al. (2009), 7880 years [15]. However, they used a date that was 8% earlier, 7 my, for the divergence time between the human and chimpanzee mtDNA lineages. We chose to retain the commonly used 6.5 my date, given the uncertainties in the phylogenetic relationships of the Miocene hominids and the amount of time that was necessary for the sorting of mtDNA lineages of the ancestral population. Nevertheless, our rate estimate is in the range of the four estimates derived by four different methods applied by Soares et al. (2009) [15], extending from 6690 to 9500 years per synonymous substitution. Notably, there is a difference in the results of the two studies that were both derived from the accumulated amount of synonymous transversions. Soares et al. (2009), however, did not correct for multiple mutation hits and, in addition, the distance was erroneously underestimated (Pedro Soares, personal communication). According to the substitution rate of 7990 years per synonymous substitution, geographically pooled sequences show that the coalescence times for the two non-African superclades, M and N, are in reasonable agreement with the earliest archaeological evidence for the presence of anatomically modern humans outside Africa (Table 6).

**Table 6:** Dates of expansion of the main clades in human mtDNA tree by different calibrations.

date of expansion (thousand years)					
clade	HVS-I (SD)^a^	coding subst. (SD)^b^	syn. transit. (SD)^c^	3^rd^ pos. (95% HPD)^d^	syn. (SD)^e^
MRCA	141 (38)	200 (25)	157 (26)	133 (78–209)	186 (31)
M	77 (16)	57 (6)	39 (5)	46 (27–71)	49 (6)
N	68 (18)	63 (6)	45 (7)	52 (30–79)	54 (8)
mean(M,N)	73	60	42	49	51

a calibration of non-coding HVS-I region (16024–16365) by Forster et al. (1996) [8], applied to the trees of Maca-Meyer et al. (2001 [63]; MRCA,N) and Kivisild et al. (2002 [64]; M).

b calibration of Mishmar et al. (2003) [65] for all coding region substitutions, applied to the Mitomap tree.

c calibration of Kivisild et al. (2006) [25] for synonymous transitions, applied to the Mitomap tree.

d data from Endicott and Ho (2008) [12], a Bayesian calibration for the 3^rd^ codon positions (interspecies).

e this study, revised calibration of Kivisild et al. (2006) [25] for synonymous substitutions, applied to the Mitomap tree.

SD - standard deviation.

HPD - highest posterior density.

The results of the present study confirm the higher non-synonymous and RNA variation in human mtDNA genealogy as compared to the level of interspecies variation [12], [14], [15], [21], [43] and reveal that the rate of accumulation of substitutions is not gradual but has accelerated since the beginning of the Holocene 11.7 kya. There are three possible explanations for the recent excessive accumulation of functional substitutions in our species: (1) adaptive shifts and positive selection, (2) insufficient time for purifying selection or (3) relaxation of selective constraints. These are not mutually exclusive and probably all of them have shaped the distribution of substitutions in human mtDNA tree. The contribution of adaptive mutations is small [25], [33]. The abundance of infrequent non-synonymous variants in human populations has been difficult to explain in terms of adaptive shift. Instead, a transient perpetuation of slightly deleterious variants [21], [22], [25] or relaxation of selection [44] have been proposed as explanations. The distinction between these two scenarios is difficult to make [23]. Comparisons to other species would enable to disentangle the effect of relaxation of natural selection due to technological, cultural and anatomical innovations of modern humans from other factors slowing down the purification of DNA from slightly deleterious mutations, such as post-glacial population growth. Indeed, various other species have been found to have higher intra-species non-synonymous variation than anticipated from interspecies differences [19], [21], [43]. There is also evidence of a delayed purifying selection after the rapid population expansion following a severe bottleneck at the LGM for the North American migratory bird species [45]. Nevertheless, because of scarcity of data, such interspecies comparisons of variation in mitochondrial genomes are so far very limited. However, the relatively high K_a_/K_s_ value of the two chimpanzee mtDNA sequences that exhibit a coalescence age of 300 ky ago implies sluggishness of purifying selection (Figure 1). The low efficiency of purifying selection may be a more general pattern as there is no sign of a rapid population growth in the genetic variation of chimpanzees [46], [47]. Demography also appears to play a role in the extent of non-synonymous variation in nuclear genes: temporal relaxation of selection after the bottleneck associated with the out-of-Africa migration has been proposed to explain the increase of probably harmful mutations from 12% in African Americans to 16% in European Americans [48]. In addition, distribution of fitness effects in a non-stationary population suggested that at least 30% of the non-synonymous or 16% of all mutations in human nuclear genes are highly deleterious [49]. An analysis of Craig Venter's exome predicted that 14% of the single nucleotide polymorphisms affect protein function [50].

It is known that published human mtDNA sequences contain sequencing errors [51], [52], [53], which may bias the results of neutrality tests [54]. Sequencing errors mostly occur on terminal branches, where no phylogenetic check or independent confirmation from another study is available. As the share of terminal branches is the largest in the youngest clades, sequencing errors can generate the accumulation of non-synonymous and RNA mutations in the youngest clades. We assessed the effect of putative sequencing errors on the results of this study by three indirect means. First, provided that the sequencing errors in the dataset are random in respect to mutation class, one can presume that the number of errors in each mutation class is proportional to the number of nucleotide positions that allow such mutations. As the number of non-synonymous positions is nearly two times higher than the number of synonymous positions, most of the randomly generated errors should appear as non-synonymous mutations. The number of positions in genes coding for RNAs is comparable to that of synonymous positions and sequencing errors are expected to occur equally likely at the two classes of nucleotide sites. However, the ratio of non-synonymous to synonymous substitutions was not elevated compared to the ratio of RNA to synonymous changes (Figure 1), suggesting no major impact of sequencing errors on our results.

**Figure 5:**
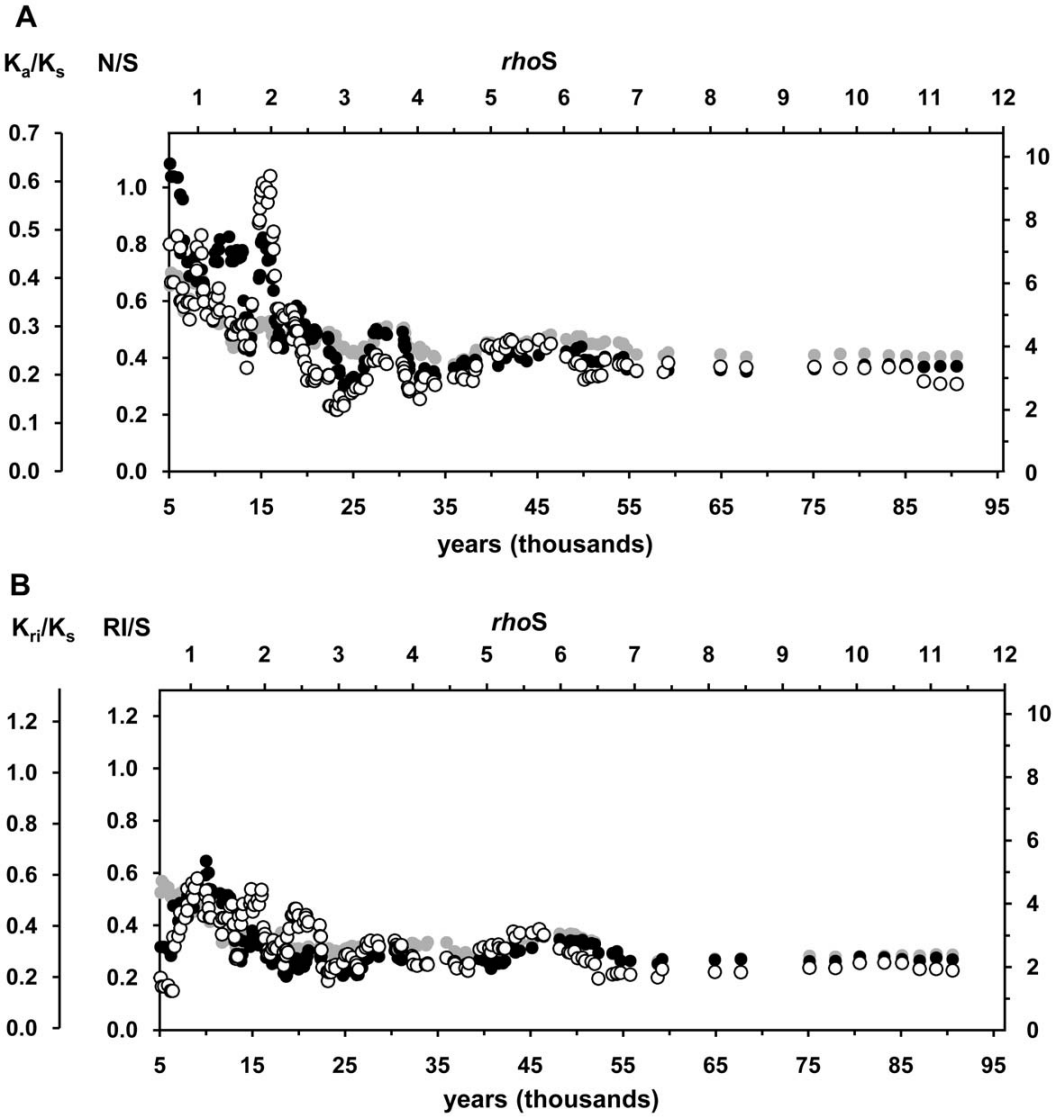
Temporal changes in the relative rates of non-synonymous (A) and RNA plus intergenic mutations (B) without counting the mutations from the branches that carried only 1 or 2 individuals. Black dots represent the results after the removal of terminal branches, open circles represent the outcome after further removal of the branches that carried 2 individuals, and grey dots show the results of the full dataset (Figure 1). The clade ages were taken from the analysis of full dataset. The upper axis (*rhoS*) gives the ages as average amount of accumulated synonymous substitutions. The lower axis shows the ages in calendar years, with 1 *rhoS* corresponding to 7990 years. The right axis gives relative changes in respect of the substitution rate inferred from the human-chimpanzee comparison. The sliding window analysis was performed as with full dataset. See the details in the text and *Methods*.

Secondly, we assessed the effect of sequencing mistakes by removing all non-synonymous and RNA mutation counts from terminal branches (and also from branches that carried 2 individuals) and repeated the sliding window analysis. This reduced the number of non-synonymous mutations in the analysis from 2065 to 605 (or 355) and the number of RNA mutations from 1407 to 442 (or 264). The observed decrease in mutation ratios was expected for all time-windows as the high ratios of external branches also contributed to the ratios of the older clades (Figure 5). After the removal of the external branches the mutation ratios were still higher for the younger clades.

Third, as phantom transversions are a common type of sequencing errors, the ratio of transversions to transitions was compared for the internal and terminal branches. The ratio for the terminal branches was 0.031 (90/2878), it was 0.033 (19/583) for branches that carried 2 individuals and 0.039 (36/929) for all others. These ratios were insignificantly different by chi-squared tests from 0.036 (55/1512), which was the average ratio for all internal branches. In conclusion, we could not find evidence of sequencing errors having major effect on our results but based on these general statistics we cannot exclude that the used dataset included minor problems of sequence quality.

We observed that the fastest evolving synonymous sites exhibited higher than expected interspecies divergence (Table 5). It is unlikely that these synonymous sites have been under strong directional selection as even for non-synonymous sites there is no evidence of strong positive selection [23], [25], [32], [36]. Alternative explanation, concerning natural selection, would be that the small historical population size of humans has reduced the efficiency of purifying selection, which enabled the mildly deleterious synonymous sites to escape the purifying selection among humans but not in chimpanzees. If the apparently fast-evolving sites were under selective pressure, the saturation hypothesis would not hold. However, the multiple hit correction, as applied on the variable rates model, was able to explain the observed gap between the intra- and interspecies synonymous substitution rates and thus we were unable to reject the null-hypothesis of neutrality. Under neutrality, an explanation would be that the mechanisms that determine the differential rate of substitution for synonymous sites are species-specific, possibly sequence context dependent, and differ in between humans and chimpanzees. Possible interspecies shifts in the synonymous substitution rates at specific sites [55] would then explain the excess of higher than expected divergence rate at sites that are also highly variable within a species. This would also further complicate the correction of observed distances for multiple hits at longer evolutionary distances as well as interspecies application of molecular clock.

In conclusion, human mitochondrial DNA clock is time-dependent mainly because of the time-dependence of purifying selection. There is also evidence that purifying selection has been counteracted by other population genetic factors during the course of human history. Our results imply that the proportion of synonymous substitutions has alternated between growth and decrease. This interpretation is strengthened given the shape of the human mtDNA tree, which reflects the bottlenecks and subsequent population expansions associated with the out-of-Africa migration and the hectic climatic conditions of the last glacial period. The wavy growth of the proportion of synonymous substitutions implies biases in the published correction of the mtDNA clock, which assumed a monotonic growth curve [15]. Therefore, the clock of synonymous substitutions should be preferred. In addition, it seems that a good consensus has been achieved on the rate of accumulation of synonymous substitutions in human mtDNA, which applies at the population as well as the interspecies level (this study, [15]).

## Methods

### Revision of the Calibration of the Molecular Clock of Synonymous Positions

The calibration of average synonymous transition rate was based on the accumulated transversions in rRNA and synonymous sites in between human and chimpanzee mtDNA lineages, assuming 6.5 my for their divergence and a constant transition/transversion rate estimated from human data [25]. Changes included 1) addition of one common chimpanzee sequence, EU095335 [56] 2) new transition/transversion ratios were calculated from the dataset that was more than 10 times the previous size [25]. The new ratio of synonymous transitions and transversions was 30.276. This estimate included the mutation counts from multiple hits presented in the phylogeny as reticulations. The ratio of synonymous transitions and rRNA transversions was 133.03. The uncorrected distances were 53.3 synonymous and 11 rRNA gene transversions to the 3 *Pan troglodytes* sequences and 51 and 10 transversions to *Pan paniscus*, respectively. The average distance from the tips to the root of the human mtDNA tree was 0.228 synonymous and 1 rRNA gene transversion. The resulting average distances were corrected by Jukes-Cantor method, accounting only for transversions and thus two thirds of all possible substitutions. The corrected interspecies distances were 53.44 synonymous and 11.54 rRNA gene transversions. The resulting substitution rate estimate is the average of the two gene classes. Altogether 4 chimpanzee sequences were used: *P. troglodytes verus* D38113 [57], *P. t. verus* X93335 or “Jenny” [58], *P. troglodytes* EU095335 [56] and *P. paniscus* NC_001644 [57]. The sequences were aligned manually in BioEdit Sequence Alignment Editor 7.0.5.3 (Tom Hall, Ibis Biosciences). For the annotation of mutations found in interspecies comparisons, we used MitoPositions, a program written in Perl that is available on request.

### The Mitomap Data

Temporal changes in the proportion of non-synonymous and RNA gene variants in human mtDNA were analyzed in 3057 sequences considering variation of coding region between positions 577 and 16023. The sequences were grouped as 183 monophyletic and 3 paraphyletic clades (Table S1). The underlying parsimonious phylogenetic tree of mtDNA coding sequences was obtained from Mitomap web page (www.mitomap.org) as of March, 2007 (Figure S1, File S1). Figure S1 shows 14 misannotations of substitutions found in the Mitomap tree. The corrections are also listed in Table S2.

The tree was transformed into a table of branch lengths and the corresponding number of individuals carried by each branch in MS Excel 2007 (Microsoft Corporation). “External branch” or “terminal branch” or “private branch” refers to such a branch that is determined by a single mtDNA sequence. Note that inclusion into this category depends on the abundance of published DNA sequences and is rather arbitrary in respect of time depth. Nucleotide substitutions were counted for the 186 clades and classified as “synonymous” (S), “non-synonymous” (N), “RNA and intergenic mutations” (RI, or RNA in the text for brevity), all of which together were termed “all substitutions” or “total variation”. Average mutational distance, *rho*, to the root haplotype of a clade was estimated [8], [59] counting only synonymous substitutions. *Rho*S values were expected to be linearly related to the clade ages.

### The Sliding Window Analysis

Temporal changes in the ratios of non-synonymous to synonymous and RNA to synonymous were assessed by sliding window analysis. The window of 15 clades was shifted by 1 along the dataset of 186 clades sorted for ascending *rhoS* values. The ratios of non-synonymous to synonymous and RNA to synonymous were calculated for each clade and the average ratio of the window was found. The median coalescence ages of the time-windows were unevenly distributed with only 7% of the ages covering half of the time span. Therefore, to avoid loss of data for the older part of the dataset, the oldest windows were allowed to run across the end of the data list with the reduction in window size. The minimum size of the last window was set to 9.

### Generation of the Reference Dataset

The empirical variation was compared to the variation of the references that otherwise resembled the empirical dataset but had uniform mutation ratios over all age groups. 10 reference datasets were generated to test for the hypothesis of age-dependent changes in the ratios of non-synonymous to synonymous or RNA to synonymous. The total number of mutations present in the empirical tree was evenly re-distributed along the branches of the empirical tree structure. The distribution of the mutations was based on binomial distribution. The fractions of the classes were expected to be 0.181 for RNA plus intergenic, 0.262 for non-synonymous and 0.557 for synonymous, as in the empirical data. Transversions were randomly selected from synonymous mutations so that they made up 3% of the synonymous mutations as in the empirical data. The reference data was subjected to sliding window analysis and went through the same processing as the empirical data. The empirical coalescence ages were used to determine the composition of the time-windows in the sliding window analysis in order to preserve the time-scale, which might have been shifted in the references with the generation of uniform mutation ratios.

### Calculation of K_a_/K_s_ and K_ri_/K_s_ Values

The non-synonymous to synonymous and RNA to synonymous mutation ratios were also presented as K_a_/K_s_ or K_ri_/K_s_ values that gave the relative rates with respect to the rate of synonymous substitutions. The number of non-synonymous sites was taken as the number of 0-fold degenerate sites or sites where all mutations result in non-synonymous amino acid change (7171). The number of synonymous sites, 4170, was taken as the sum over all 4-fold (2026) and 2-fold (2144) degenerate sites. The number of RNA plus intergenic sites was 4106 (4017 RNA+89 intergenic). We note that the low occurrence of transversions justifies the counting of 2-fold sites as synonymous sites. Only 1.9% (41/2144) of the substitutions at the 2-fold degenerate sites were transversions (and thus non-synonymous) in the Mitomap genealogy and we counted only 2 transversions at 2-fold sites in the alignment of rCRS [40] and chimpanzee sequences [56].

### The Substitution Rate Over All Coding Sites

The overall rate was derived from the ratio of all substitutions to synonymous substitutions. It was calculated window-wise as the sum of non-synonymous to synonymous ratio + RNA to synonymous ratio + 1. The basic rate was determined by the human-chimpanzee comparisons. Notably, the average K_a_/K_s_ for the human-chimp distance, derived from several ML estimations by Green et al. (2008) [60], was 0.0685, which would equal to a non-synonymous to synonymous ratio of 0.118. This value was equal to the non-synonymous to synonymous ratio of 0.118 (188/1594), which we obtained with the Jukes-Cantor correction for the human-chimp distance in non-synonymous mutations and our estimation of the distance for synonymous mutations with the 3-rate model (see *Results*). However, we chose a different chimp sequence, EU095335 [56] instead of NC_001643 [57]. This yielded a K_a_/K_s_ of 0.065 and non-synonymous to synonymous ratio of 0.112. Analogously, the K_ri_/K_s_ value was found to be 0.124 and the respective RNA to synonymous ratio was 0.122.

## Supporting Information

File S1 The bibliography of the Mitomap tree from www.mitomap.org.
(0.10 MB DOC)

Figure S1 Mitomap tree. Phylogenetic tree of human mitochondrial DNA coding region (positions 577-16023) sequences from Mitomap web page as of March, 2007 (www.mitomap.org). Inside the figure the legend explains the color code for the substitution classes. Identified mis-assignments (Table S2) have been underlined and the corrections maintaining the original color scheme have been added to the figure.
(0.50 MB PDF)

Table S1 Mutation counts and coalescence ages of the 186 analyzed clades.
(0.33 MB DOC)

Table S2 Corrected misannotations of the mutations on the Mitomap tree.
(0.05 MB DOC)
