# Supplementary material for: Explaining the Imperfection of the Molecular Clock of Hominid Mitochondria
Source: PLoS One. 2009 Dec 29;4(12):e8260. doi: 10.1371/journal.pone.0008260 (PMC2794369; doi:10.1371/journal.pone.0008260)
Supplement: File S1 — The bibliography of the Mitomap tree from www.mitomap.org. (0.10 MB DOC) [file pone.0008260.s001.doc]

**Bibliography for Mitomap's** [**Phylogenetic Tree**](http://www.mitomap.org/mitomap-phylogeny.pdf) **
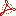
**

9/25/2006

1, [Mishmar D, Ruiz-Pesini E, Golik P, Macaulay V, Clark AG, Hosseini S, Brandon M, Easley K, Chen E, Brown MD, Sukernik RI, Olckers A, Wallace DC.](http://www.ncbi.nlm.nih.gov/entrez/query.fcgi?db=pubmed&cmd=Retrieve&dopt=Citation&list_uids=12509511&query_hl=4&itool=pubmed_docsum) Natural selection shaped regional mtDNA variation in humans. Proc Natl Acad Sci U S A. 2003 Jan 7;100(1):171-6. Epub 2002 Dec 30. PMID: 12509511

2, Procaccio et al, Unpublished

3, [Ingman M, Kaessmann H, Paabo S, Gyllensten U.](http://www.ncbi.nlm.nih.gov/entrez/query.fcgi?db=pubmed&cmd=Retrieve&dopt=Citation&list_uids=11130070&query_hl=6&itool=pubmed_docsum) Mitochondrial genome variation and the origin of modern humans. Nature. 2000 Dec 7;408(6813):708-13. Erratum in: Nature 2001 Mar 29;410(6828):611. PMID: 11130070

4, [Herrnstadt C, Elson JL, Fahy E, Preston G, Turnbull DM, Anderson C, Ghosh SS, Olefsky JM, Beal MF, Davis RE, Howell N.](http://www.ncbi.nlm.nih.gov/entrez/query.fcgi?db=pubmed&cmd=Retrieve&dopt=Citation&list_uids=11938495&query_hl=7&itool=pubmed_docsum) Reduced-median-network analysis of complete mitochondrial DNA coding-region sequences for the major African, Asian, and European haplogroups. Am J Hum Genet. 2002 May;70(5):1152-71. Epub 2002 Apr 5. Erratum in: Am J Hum Genet 2002 Aug;71(2):448-9. PMID: 11938495

5, [Horai S, Hayasaka K, Kondo R, Tsugane K, Takahata N.](http://www.ncbi.nlm.nih.gov/entrez/query.fcgi?db=pubmed&cmd=Retrieve&dopt=Citation&list_uids=7530363&query_hl=8&itool=pubmed_docsum) Recent African origin of modern humans revealed by complete sequences of hominoid mitochondrial DNAs. Proc Natl Acad Sci U S A. 1995 Jan 17;92(2):532-6. PMID: 7530363

6, [Torroni A, Rengo C, Guida V, Cruciani F, Sellitto D, Coppa A, Calderon FL, Simionati B, Valle G, Richards M, Macaulay V, Scozzari R.](http://www.ncbi.nlm.nih.gov/entrez/query.fcgi?db=pubmed&cmd=Retrieve&dopt=Citation&list_uids=11595973&query_hl=9&itool=pubmed_docsum) Do the four clades of the mtDNA haplogroup L2 evolve at different rates? Am J Hum Genet. 2001 Dec;69(6):1348-56. Epub 2001 Oct 10. PMID: 11595973

7, [Maca-Meyer N, Gonzalez AM, Larruga JM, Flores C, Cabrera VM.](http://www.ncbi.nlm.nih.gov/entrez/query.fcgi?db=pubmed&cmd=Retrieve&dopt=Citation&list_uids=11553319&query_hl=10&itool=pubmed_docsum) Major genomic mitochondrial lineages delineate early human expansions. BMC Genet. 2001;2:13. Epub 2001 Aug 13. PMID: 11553319

8, [Nishino I, Seki A, Maegaki Y, Takeshita K, Horai S, Nonaka I, Goto Y.](http://www.ncbi.nlm.nih.gov/entrez/query.fcgi?db=pubmed&cmd=Retrieve&dopt=Citation&list_uids=8769114&query_hl=12&itool=pubmed_docsum) A novel mutation in the mitochondrial tRNA(Thr) gene associated with a mitochondrial encephalomyopathy. Biochem Biophys Res Commun. 1996 Aug 5;225(1):180-5. PMID: 8769114

9, [Finnila S, Lehtonen MS, Majamaa K.](http://www.ncbi.nlm.nih.gov/entrez/query.fcgi?db=pubmed&cmd=Retrieve&dopt=Citation&list_uids=11349229&query_hl=15&itool=pubmed_docsum) Phylogenetic network for European mtDNA. Am J Hum Genet. 2001 Jun;68(6):1475-84. Epub 2001 May 10. PMID: 11349229

10, [Derbeneva OA, Sukernik RI, Volodko NV, Hosseini SH, Lott MT, Wallace DC.](http://www.ncbi.nlm.nih.gov/entrez/query.fcgi?db=pubmed&cmd=Retrieve&dopt=Citation&list_uids=12082644&query_hl=16&itool=pubmed_docsum) Analysis of mitochondrial DNA diversity in the aleuts of the commander islands and its implications for the genetic history of beringia. Am J Hum Genet. 2002 Aug;71(2):415-21. Epub 2002 Jun 25. PMID: 12082644

11, [Ozawa T, Tanaka M, Ino H, Ohno K, Sano T, Wada Y, Yoneda M, Tanno Y, Miyatake T, Tanaka T, et al.](http://www.ncbi.nlm.nih.gov/entrez/query.fcgi?db=pubmed&cmd=Retrieve&dopt=Citation&list_uids=2025303&query_hl=19&itool=pubmed_docsum) Distinct clustering of point mutations in mitochondrial DNA among patients with mitochondrial encephalomyopathies and with Parkinson's disease. Biochem Biophys Res Commun. 1991 Apr 30;176(2):938-46. PMID: 2025303

12, (Data removed from tree due to poor quality of sequences)

13, [Ozawa T.](http://www.ncbi.nlm.nih.gov/entrez/query.fcgi?db=pubmed&cmd=Retrieve&dopt=Citation&list_uids=7556507&query_hl=22&itool=pubmed_DocSum) Mechanism of somatic mitochondrial DNA mutations associated with age and diseases. Biochim Biophys Acta. 1995 May 24;1271(1):177-89. Review. PMID: 7599206

14, [Ikebe S, Tanaka M, Ozawa T.](http://www.ncbi.nlm.nih.gov/entrez/query.fcgi?db=pubmed&cmd=Retrieve&dopt=Citation&list_uids=7723627&query_hl=30&itool=pubmed_docsum) Point mutations of mitochondrial genome in Parkinson's disease. Brain Res Mol Brain Res. 1995 Feb;28(2):281-95. PMID: 7723627

15, (Data removed from tree due to poor quality of sequences)

16, [Andrews RM, Kubacka I, Chinnery PF, Lightowlers RN, Turnbull DM, Howell N.](http://www.ncbi.nlm.nih.gov/entrez/query.fcgi?db=pubmed&cmd=Retrieve&dopt=Citation&list_uids=10508508&query_hl=32&itool=pubmed_docsum) Reanalysis and revision of the Cambridge reference sequence for human mitochondrial DNA. Nat Genet. 1999 Oct;23(2):147. No abstract available. PMID: 10508508

17, [Leo-Kottler B, Luberichs J, Besch D, Christ-Adler M, Fauser S.](http://www.ncbi.nlm.nih.gov/entrez/query.fcgi?db=pubmed&cmd=Retrieve&dopt=Citation&list_uids=12271374&query_hl=38&itool=pubmed_docsum) Leber's hereditary optic neuropathy: clinical and molecular genetic results in a patient with a point mutation at np T11253C (isoleucine to threonine) in the ND4 gene and spontaneous recovery. Graefes Arch Clin Exp Ophthalmol. 2002 Sep;240(9):758-64. Epub 2002 Aug 2. PMID: 12271374

18, [Arnason U, Xu X, Gullberg A.](http://www.ncbi.nlm.nih.gov/entrez/query.fcgi?db=pubmed&cmd=Retrieve&dopt=Citation&list_uids=8919866&query_hl=41&itool=pubmed_docsum) Comparison between the complete mitochondrial DNA sequences of Homo and the common chimpanzee based on nonchimeric sequences. J Mol Evol. 1996 Feb;42(2):145-52. PMID: 8919866

19, [Shin WS, Tanaka M, Suzuki J, Hemmi C, Toyo-oka T.](http://www.ncbi.nlm.nih.gov/entrez/query.fcgi?db=pubmed&cmd=Retrieve&dopt=Citation&list_uids=11038324&query_hl=43&itool=pubmed_docsum) A novel homoplasmic mutation in mtDNA with a single evolutionary origin as a risk factor for cardiomyopathy. Am J Hum Genet. 2000 Dec;67(6):1617-20. Epub 2000 Oct 18. PMID: 11038324 (see also [Kivisild T, Tolk HV, Parik J, Wang Y, Papiha SS, Bandelt HJ, Villems R.](http://www.ncbi.nlm.nih.gov/entrez/query.fcgi?db=pubmed&cmd=Retrieve&dopt=Citation&list_uids=12270900&query_hl=45&itool=pubmed_docsum) The emerging limbs and twigs of the East Asian mtDNA tree. Mol Biol Evol. 2002 Oct;19(10):1737-51. Erratum in: Mol Biol Evol. 2003 Jan;20(1):162. PMID: 12270900 )

20, (Data removed from tree due to poor quality of sequences)

21, (Data removed from tree due to poor quality of sequences)

22, [Schwartz F, Baldwin CT, Baima J, Gavras H.](http://www.ncbi.nlm.nih.gov/entrez/query.fcgi?db=pubmed&cmd=Retrieve&dopt=Citation&list_uids=10449650&query_hl=46&itool=pubmed_docsum) Mitochondrial DNA mutations in patients with orthostatic hypotension. Am J Med Genet. 1999 Sep 10;86(2):145-50. PMID: 10449650

23, [Fauser S, Luberichs J, Besch D, Leo-Kottler B.](http://www.ncbi.nlm.nih.gov/entrez/query.fcgi?db=pubmed&cmd=Retrieve&dopt=Citation&list_uids=12150954&query_hl=47&itool=pubmed_docsum) Sequence analysis of the complete mitochondrial genome in patients with Leber's hereditary optic neuropathy lacking the three most common pathogenic DNA mutations. Biochem Biophys Res Commun. 2002 Jul 12;295(2):342-7. PMID: 12150954

24, [Brown MD, Zhadanov S, Allen JC, Hosseini S, Newman NJ, Atamonov VV, Mikhailovskaya IE, Sukernik RI, Wallace DC.](http://www.ncbi.nlm.nih.gov/entrez/query.fcgi?db=pubmed&cmd=Retrieve&dopt=Citation&list_uids=11479733&query_hl=49&itool=pubmed_docsum) Novel mtDNA mutations and oxidative phosphorylation dysfunction in Russian LHON families. Hum Genet. 2001 Jul;109(1):33-9. PMID: 11479733

25, [Brown MD, Starikovskaya E, Derbeneva O, Hosseini S, Allen JC, Mikhailovskaya IE, Sukernik RI, Wallace DC.](http://www.ncbi.nlm.nih.gov/entrez/query.fcgi?db=pubmed&cmd=Retrieve&dopt=Citation&list_uids=11935318&query_hl=50&itool=pubmed_docsum) The role of mtDNA background in disease expression: a new primary LHON mutation associated with Western Eurasian haplogroup J. Hum Genet. 2002 Feb;110(2):130-8. Epub 2002 Jan 24. PMID: 11935318

26, [McMahon FJ, Chen YS, Patel S, Kokoszka J, Brown MD, Torroni A, DePaulo JR, Wallace DC.](http://www.ncbi.nlm.nih.gov/entrez/query.fcgi?db=pubmed&cmd=Retrieve&dopt=Citation&list_uids=10873911&query_hl=56&itool=pubmed_docsum) Mitochondrial DNA sequence diversity in bipolar affective disorder. Am J Psychiatry. 2000 Jul;157(7):1058-64. PMID: 10873911

27, [Yamasoba T, Goto Y, Oka Y, Nishino I, Tsukuda K, Nonaka I.](http://www.ncbi.nlm.nih.gov/entrez/query.fcgi?db=pubmed&cmd=Retrieve&dopt=Citation&list_uids=12031626&query_hl=58&itool=pubmed_docsum) Atypical muscle pathology and a survey of cis-mutations in deaf patients harboring a 1555 A-to-G point mutation in the mitochondrial ribosomal RNA gene. Neuromuscul Disord. 2002 Jun;12(5):506-12. PMID: 12031626

28, [Kim JY, Hwang JM, Park SS.](http://www.ncbi.nlm.nih.gov/entrez/query.fcgi?db=pubmed&cmd=Retrieve&dopt=Citation&list_uids=12112111&query_hl=61&itool=pubmed_docsum) Mitochondrial DNA C4171A/ND1 is a novel primary causative mutation of Leber's hereditary optic neuropathy with a good prognosis. Ann Neurol. 2002 May;51(5):630-4. PMID: 12112111

29, [Rose G, Passarino G, Carrieri G, Altomare K, Greco V, Bertolini S, Bonafe M, Franceschi C, De Benedictis G.](http://www.ncbi.nlm.nih.gov/entrez/query.fcgi?db=pubmed&cmd=Retrieve&dopt=Citation&list_uids=11571560&query_hl=69&itool=pubmed_docsum) Paradoxes in longevity: sequence analysis of mtDNA haplogroup J in centenarians. Eur J Hum Genet. 2001 Sep;9(9):701-7. PMID: 11571560

30, [Howell N, Miller NR, Mackey DA, Arnold A, Herrnstadt C, Williams IM, Kubacka I.](http://www.ncbi.nlm.nih.gov/entrez/query.fcgi?db=pubmed&cmd=Retrieve&dopt=Citation&list_uids=12464729&query_hl=71&itool=pubmed_docsum) Lightning strikes twice: Leber hereditary optic neuropathy families with two pathogenic mtDNA mutations. J Neuroophthalmol. 2002 Dec;22(4):262-9. PMID: 12464729

31, [Ohlenbusch A, Wilichowski E, Hanefeld F.](http://www.ncbi.nlm.nih.gov/entrez/query.fcgi?db=pubmed&cmd=Retrieve&dopt=Citation&list_uids=9762692&query_hl=76&itool=pubmed_docsum) Characterization of the mitochondrial genome in childhood multiple sclerosis. I. Optic neuritis and LHON mutations. Neuropediatrics. 1998 Aug;29(4):175-9. PMID: 9762692

32, [Mimaki M, Ikota A, Sato A, Komaki H, Akanuma J, Nonaka I, Goto Y.](http://www.ncbi.nlm.nih.gov/entrez/query.fcgi?db=pubmed&cmd=Retrieve&dopt=Citation&list_uids=12560876&query_hl=79&itool=pubmed_docsum) A double mutation (G11778A and G12192A) in mitochondrial DNA associated with Leber's hereditary optic neuropathy and cardiomyopathy. J Hum Genet. 2003;48(1):47-50. PMID: 12560876

33, [Howell N, Smejkal CB, Mackey DA, Chinnery PF, Turnbull DM, Herrnstadt C.](http://www.ncbi.nlm.nih.gov/entrez/query.fcgi?db=pubmed&cmd=Retrieve&dopt=Citation&list_uids=12571803&query_hl=250&itool=pubmed_docsum) The pedigree rate of sequence divergence in the human mitochondrial genome: there is a difference between phylogenetic and pedigree rates. Am J Hum Genet. 2003 Mar;72(3):659-70. Epub 2003 Feb 4. PMID: 12571803

34, [Kalman B, Lublin FD, Alder H.](http://www.ncbi.nlm.nih.gov/entrez/query.fcgi?db=pubmed&cmd=Retrieve&dopt=Citation&list_uids=8866430&query_hl=171&itool=pubmed_docsum) Characterization of the mitochondrial DNA in patients with multiple sclerosis. J Neurol Sci. 1996 Sep 1;140(1-2):75-84. PMID: 8866430

35, [Ozawa T, Tanaka M, Sugiyama S, Ino H, Ohno K, Hattori K, Ohbayashi T, Ito T, Deguchi H, Kawamura K, et al.](http://www.ncbi.nlm.nih.gov/entrez/query.fcgi?db=pubmed&cmd=Retrieve&dopt=Citation&list_uids=2043137&query_hl=19&itool=pubmed_docsum) Patients with idiopathic cardiomyopathy belong to the same mitochondrial DNA gene family of Parkinson's disease and mitochondrial encephalomyopathy. Biochem Biophys Res Commun. 1991 May 31;177(1):518-25. PMID: 2043137

36, (Data removed from tree due to poor quality of sequences)

37, [Chinnery PF, Brown DT, Andrews RM, Singh-Kler R, Riordan-Eva P, Lindley J, Applegarth DA, Turnbull DM, Howell N.](http://www.ncbi.nlm.nih.gov/entrez/query.fcgi?db=pubmed&cmd=Retrieve&dopt=Citation&list_uids=11133798&query_hl=153&itool=pubmed_docsum) The mitochondrial ND6 gene is a hot spot for mutations that cause Leber's hereditary optic neuropathy. Brain. 2001 Jan;124(Pt 1):209-18. PMID: 11133798

38, [Brown MD, Voljavec AS, Lott MT, MacDonald I, Wallace DC.](http://www.ncbi.nlm.nih.gov/entrez/query.fcgi?db=pubmed&cmd=Retrieve&dopt=Citation&list_uids=1634041&query_hl=154&itool=pubmed_docsum) Leber's hereditary optic neuropathy: a model for mitochondrial neurodegenerative diseases. FASEB J. 1992 Jul;6(10):2791-9. Review. PMID: 1634041

39, [Lindholm E, Cavelier L, Howell WM, Eriksson I, Jalonen P, Adolfsson R, Blackwood DH, Muir WJ, Brookes AJ, Gyllensten U, Jazin EE.](http://www.ncbi.nlm.nih.gov/entrez/query.fcgi?db=pubmed&cmd=Retrieve&dopt=Citation&list_uids=9450186&query_hl=158&itool=pubmed_docsum) Mitochondrial sequence variants in patients with schizophrenia. Eur J Hum Genet. 1997 Nov-Dec;5(6):406-12. PMID: 9450186

40, [Malik S, Sudoyo H, Sasmono T, Winata S, Arhya IN, Pramoonjago P, Sudana W, Marzuki S.](http://www.ncbi.nlm.nih.gov/entrez/query.fcgi?db=pubmed&cmd=Retrieve&dopt=Citation&list_uids=12624722&query_hl=159&itool=pubmed_docsum) Nonsyndromic sensorineural deafness associated with the A1555G mutation in the mitochondrial small subunit ribosomal RNA in a Balinese family. J Hum Genet. 2003;48(3):119-24. PMID: 12624722

41, [Howell N, Oostra RJ, Bolhuis PA, Spruijt L, Clarke LA, Mackey DA, Preston G, Herrnstadt C.](http://www.ncbi.nlm.nih.gov/entrez/query.fcgi?db=pubmed&cmd=Retrieve&dopt=Citation&list_uids=12736867&query_hl=80&itool=pubmed_docsum) Sequence analysis of the mitochondrial genomes from Dutch pedigrees with Leber hereditary optic neuropathy. Am J Hum Genet. 2003 Jun;72(6):1460-9. Epub 2003 May 6. PMID: 12736867

42, [Howell N, Herrnstadt C, Shults C, Mackey DA.](http://www.ncbi.nlm.nih.gov/entrez/query.fcgi?db=pubmed&cmd=Retrieve&dopt=Citation&list_uids=12749053&query_hl=250&itool=pubmed_docsum) Low penetrance of the 14484 LHON mutation when it arises in a non-haplogroup J mtDNA background. Am J Med Genet A. 2003 Jun 1;119(2):147-51. PMID: 12749053

43, [Lehtonen MS, Moilanen JS, Majamaa K.](http://www.ncbi.nlm.nih.gov/entrez/query.fcgi?db=pubmed&cmd=Retrieve&dopt=Citation&list_uids=12802679&query_hl=167&itool=pubmed_docsum) Increased variation in mtDNA in patients with familial sensorineural hearing impairment. Hum Genet. 2003 Aug;113(3):220-7. Epub 2003 Jun 12. PMID: 12802679

44, [Montiel-Sosa F, Ruiz-Pesini E, Enriquez JA, Marcuello A, Diez-Sanchez C, Montoya J, Wallace DC, Lopez-Perez MJ.](http://www.ncbi.nlm.nih.gov/entrez/query.fcgi?db=pubmed&cmd=Retrieve&dopt=Citation&list_uids=16326035&query_hl=166&itool=pubmed_docsum) Differences of sperm motility in mitochondrial DNA haplogroup U sublineages. Gene. 2006 Mar 1;368:21-7. Epub 2005 Dec 1. PMID: 16326035

45, [Ingman M, Gyllensten U.](http://www.ncbi.nlm.nih.gov/entrez/query.fcgi?db=pubmed&cmd=Retrieve&dopt=Citation&list_uids=12840039&query_hl=164&itool=pubmed_docsum) Mitochondrial genome variation and evolutionary history of Australian and New Guinean aborigines. Genome Res. 2003 Jul;13(7):1600-6. PMID: 12840039

46, [Kong QP, Yao YG, Sun C, Bandelt HJ, Zhu CL, Zhang YP.](http://www.ncbi.nlm.nih.gov/entrez/query.fcgi?db=pubmed&cmd=Retrieve&dopt=Citation&list_uids=12870132&query_hl=162&itool=pubmed_docsum) Phylogeny of east Asian mitochondrial DNA lineages inferred from complete sequences. Am J Hum Genet. 2003 Sep;73(3):671-6. Epub 2003 Jul 17. Erratum in: Am J Hum Genet. 2004 Jun;75(1):157. PMID: 12870132

47, [Starikovskaya EB, Sukernik RI, Derbeneva OA, Volodko NV, Ruiz-Pesini E, Torroni A, Brown MD, Lott MT, Hosseini SH, Huoponen K, Wallace DC.](http://www.ncbi.nlm.nih.gov/entrez/query.fcgi?db=pubmed&cmd=Retrieve&dopt=Citation&list_uids=15638829&query_hl=161&itool=pubmed_docsum) Mitochondrial DNA diversity in indigenous populations of the southern extent of Siberia, and the origins of Native American haplogroups. Ann Hum Genet. 2005 Jan;69(Pt 1):67-89. PMID: 15638829

48, [Maca-Meyer N, Gonzalez AM, Pestano J, Flores C, Larruga JM, Cabrera VM.](http://www.ncbi.nlm.nih.gov/entrez/query.fcgi?db=pubmed&cmd=Retrieve&dopt=Citation&list_uids=14563219&query_hl=160&itool=pubmed_docsum) Mitochondrial DNA transit between West Asia and North Africa inferred from U6 phylogeography. BMC Genet. 2003 Oct 16;4:15. PMID: 14563219

49, [Reidla M, Kivisild T, Metspalu E, Kaldma K, Tambets K, Tolk HV, Parik J, Loogvali EL, Derenko M, Malyarchuk B, Bermisheva M, Zhadanov S, Pennarun E, Gubina M, Golubenko M, Damba L, Fedorova S, Gusar V, Grechanina E, Mikerezi I, Moisan JP, Chaventre A, Khusnutdinova E, Osipova L, Stepanov V, Voevoda M, Achilli A, Rengo C, Rickards O, De Stefano GF, Papiha S, Beckman L, Janicijevic B, Rudan P, Anagnou N, Michalodimitrakis E, Koziel S, Usanga E, Geberhiwot T, Herrnstadt C, Howell N, Torroni A, Villems R.](http://www.ncbi.nlm.nih.gov/entrez/query.fcgi?db=pubmed&cmd=Retrieve&dopt=Citation&list_uids=14574647&query_hl=152&itool=pubmed_docsum) Origin and diffusion of mtDNA haplogroup X. Am J Hum Genet. 2003 Nov;73(5):1178-90. Epub 2003 Oct 20. PMID: 14574647

50, [Levin BC, Cheng H, Reeder DJ.](http://www.ncbi.nlm.nih.gov/entrez/query.fcgi?db=pubmed&cmd=Retrieve&dopt=Citation&list_uids=9933560&query_hl=1&itool=pubmed_docsum) A human mitochondrial DNA standard reference material for quality control in forensic identification, medical diagnosis, and mutation detection. Genomics. 1999 Jan 15;55(2):135-46. PMID: 9933560

51, [Bandelt HJ, Herrnstadt C, Yao YG, Kong QP, Kivisild T, Rengo C, Scozzari R, Richards M, Villems R, Macaulay V, Howell N, Torroni A, Zhang YP.](http://www.ncbi.nlm.nih.gov/entrez/query.fcgi?db=pubmed&cmd=Retrieve&dopt=Citation&list_uids=14641239&query_hl=147&itool=pubmed_docsum) Identification of Native American founder mtDNAs through the analysis of complete mtDNA sequences: some caveats. Ann Hum Genet. 2003 Nov;67(Pt 6):512-24. PMID: 14641239

52, Mouton et al, Unpublished

53, [Brown MD, Hosseini S, Steiner I, Wallace DC, Korn-Lubetzki I.](http://www.ncbi.nlm.nih.gov/entrez/query.fcgi?db=pubmed&cmd=Retrieve&dopt=Citation&list_uids=14978686&query_hl=145&itool=pubmed_DocSum) Complete mitochondrial DNA sequence analysis in a family with early-onset dystonia and optic atrophy. Mov Disord. 2004 Feb;19(2):235-7. PMID: 14978686

54, Procaccio et al, Unpublished

55, Ruiz-Pesini et al, Unpublished

56, Procaccio et al, Unpublished

57, [Tanaka M, Takeyasu T, Fuku N, Li-Jun G, Kurata M.](http://www.ncbi.nlm.nih.gov/entrez/query.fcgi?db=pubmed&cmd=Retrieve&dopt=Citation&list_uids=15126279&query_hl=141&itool=pubmed_docsum) Mitochondrial genome single nucleotide polymorphisms and their phenotypes in the Japanese. Ann N Y Acad Sci. 2004 Apr;1011:7-20. PMID: 15126279

58, [Palanichamy MG, Sun C, Agrawal S, Bandelt HJ, Kong QP, Khan F, Wang CY, Chaudhuri TK, Palla V, Zhang YP.](http://www.ncbi.nlm.nih.gov/entrez/query.fcgi?db=pubmed&cmd=Retrieve&dopt=Citation&list_uids=15467980&query_hl=138&itool=pubmed_docsum) Phylogeny of mitochondrial DNA macrohaplogroup N in India, based on complete sequencing: implications for the peopling of South Asia. Am J Hum Genet. 2004 Dec;75(6):966-78. Epub 2004 Oct 1. PMID: 15467980

59, [Achilli A, Rengo C, Magri C, Battaglia V, Olivieri A, Scozzari R, Cruciani F, Zeviani M, Briem E, Carelli V, Moral P, Dugoujon JM, Roostalu U, Loogvali EL, Kivisild T, Bandelt HJ, Richards M, Villems R, Santachiara-Benerecetti AS, Semino O, Torroni A.](http://www.ncbi.nlm.nih.gov/entrez/query.fcgi?db=pubmed&cmd=Retrieve&dopt=Citation&list_uids=15382008&query_hl=120&itool=pubmed_docsum) The molecular dissection of mtDNA haplogroup H confirms that the Franco-Cantabrian glacial refuge was a major source for the European gene pool. Am J Hum Genet. 2004 Nov;75(5):910-8. Epub 2004 Sep 20. PMID: 15382008

60, [Uusimaa J, Finnila S, Remes AM, Rantala H, Vainionpaa L, Hassinen IE, Majamaa K.](http://www.ncbi.nlm.nih.gov/entrez/query.fcgi?db=pubmed&cmd=Retrieve&dopt=Citation&list_uids=15286228&query_hl=135&itool=pubmed_docsum) Molecular epidemiology of childhood mitochondrial encephalomyopathies in a Finnish population: sequence analysis of entire mtDNA of 17 children reveals heteroplasmic mutations in tRNA^Arg^, tRNA^Glu^, and tRNA^Leu(UUR)^ genes. Pediatrics. 2004 Aug;114(2):443-50. PMID: 15286228

61, [Da Pozzo P, Cardaioli E, Radi E, Federico A.](http://www.ncbi.nlm.nih.gov/entrez/query.fcgi?db=pubmed&cmd=Retrieve&dopt=Citation&list_uids=15465027&query_hl=134&itool=pubmed_docsum) Sequence analysis of the complete mitochondrial genome in patients with mitochondrial encephaloneuromyopathies lacking the common pathogenic DNA mutations. Biochem Biophys Res Commun. 2004 Nov 5;324(1):360-4. PMID: 15465027

62, [Howell N, Elson JL, Turnbull DM, Herrnstadt C.](http://www.ncbi.nlm.nih.gov/entrez/query.fcgi?db=pubmed&cmd=Retrieve&dopt=Citation&list_uids=15190127&query_hl=131&itool=pubmed_docsum) African Haplogroup L mtDNA sequences show violations of clock-like evolution. Mol Biol Evol. 2004 Oct;21(10):1843-54. Epub 2004 Jun 9. PMID: 15190127

63, [Coble MD, Just RS, O'Callaghan JE, Letmanyi IH, Peterson CT, Irwin JA, Parsons TJ.](http://www.ncbi.nlm.nih.gov/entrez/query.fcgi?db=pubmed&cmd=Retrieve&dopt=Citation&list_uids=14760490&query_hl=129&itool=pubmed_docsum) Single nucleotide polymorphisms over the entire mtDNA genome that increase the power of forensic testing in Caucasians. Int J Legal Med. 2004 Jun;118(3):137-46. Epub 2004 Feb 4. PMID: 14760490

64, Coskun et al, Unpublished

65, [Wang Q, Li R, Zhao H, Peters JL, Liu Q, Yang L, Han D, Greinwald JH Jr, Young WY, Guan MX.](http://www.ncbi.nlm.nih.gov/entrez/query.fcgi?db=pubmed&cmd=Retrieve&dopt=Citation&list_uids=15637703&query_hl=350&itool=pubmed_docsum) Clinical and molecular characterization of a Chinese patient with auditory neuropathy associated with mitochondrial 12S rRNA T1095C mutation. Am J Med Genet A. 2005 Feb 15;133(1):27-30. PMID: 15637703

66, [Zhao L, Young WY, Li R, Wang Q, Qian Y, Guan MX.](http://www.ncbi.nlm.nih.gov/entrez/query.fcgi?db=pubmed&cmd=Retrieve&dopt=Citation&list_uids=15555598&query_hl=125&itool=pubmed_docsum) Clinical evaluation and sequence analysis of the complete mitochondrial genome of three Chinese patients with hearing impairment associated with the 12S rRNA T1095C mutation. Biochem Biophys Res Commun. 2004 Dec 24;325(4):1503-8. PMID: 15555598

67, [Trejaut JA, Kivisild T, Loo JH, Lee CL, He CL, Hsu CJ, Lee ZY, Lin M.](http://www.ncbi.nlm.nih.gov/entrez/query.fcgi?db=pubmed&cmd=Retrieve&dopt=Citation&list_uids=15984912&query_hl=122&itool=pubmed_docsum) Traces of archaic mitochondrial lineages persist in Austronesian-speaking Formosan populations. PLoS Biol. 2005 Aug;3(8):e247. Epub 2005 Jul 5. Erratum in: PLoS Biol. 2005 Oct;3(10):e376. Li, Zheng Yuan [corrected to Lee, Zheng Yuan]. PMID: 15984912

68, [Achilli A, Rengo C, Battaglia V, Pala M, Olivieri A, Fornarino S, Magri C, Scozzari R, Babudri N, Santachiara-Benerecetti AS, Bandelt HJ, Semino O, Torroni A.](http://www.ncbi.nlm.nih.gov/entrez/query.fcgi?db=pubmed&cmd=Retrieve&dopt=Citation&list_uids=15791543&query_hl=120&itool=pubmed_docsum) Saami and Berbers--an unexpected mitochondrial DNA link. Am J Hum Genet. 2005 May;76(5):883-6. Epub 2005 Mar 24. PMID: 15791543

69, [Rajkumar R, Banerjee J, Gunturi HB, Trivedi R, Kashyap VK.](http://www.ncbi.nlm.nih.gov/entrez/query.fcgi?db=pubmed&cmd=Retrieve&dopt=Citation&list_uids=15804362&query_hl=119&itool=pubmed_docsum) Phylogeny and antiquity of M macrohaplogroup inferred from complete mt DNA sequence of Indian specific lineages. BMC Evol Biol. 2005 Apr 2;5(1):26. Erratum in: BMC Evol Biol. 2006;6(9):9. PMID: 15804362

70, [Friedlaender J, Schurr T, Gentz F, Koki G, Friedlaender F, Horvat G, Babb P, Cerchio S, Kaestle F, Schanfield M, Deka R, Yanagihara R, Merriwether DA.](http://www.ncbi.nlm.nih.gov/entrez/query.fcgi?db=pubmed&cmd=Retrieve&dopt=Citation&list_uids=15814828&query_hl=332&itool=pubmed_docsum) Expanding Southwest Pacific mitochondrial haplogroups P and Q. Mol Biol Evol. 2005 Jun;22(6):1506-17. Epub 2005 Apr 6. Erratum in: Mol Biol Evol. 2005 Nov;22(11):2313. PMID: 15814828

71, [Macaulay V, Hill C, Achilli A, Rengo C, Clarke D, Meehan W, Blackburn J, Semino O, Scozzari R, Cruciani F, Taha A, Shaari NK, Raja JM, Ismail P, Zainuddin Z, Goodwin W, Bulbeck D, Bandelt HJ, Oppenheimer S, Torroni A, Richards M.](http://www.ncbi.nlm.nih.gov/entrez/query.fcgi?db=pubmed&cmd=Retrieve&dopt=Citation&list_uids=15890885&query_hl=114&itool=pubmed_docsum) Single, rapid coastal settlement of Asia revealed by analysis of complete mitochondrial genomes. Science. 2005 May 13;308(5724):1034-6. PMID: 15890885

72, [Thangaraj K, Chaubey G, Kivisild T, Reddy AG, Singh VK, Rasalkar AA, Singh L.](http://www.ncbi.nlm.nih.gov/entrez/query.fcgi?db=pubmed&cmd=Retrieve&dopt=Citation&list_uids=15890876&query_hl=111&itool=pubmed_docsum) Reconstructing the origin of Andaman Islanders. Science. 2005 May 13;308(5724):996. PMID: 15890876

73, [Zsurka G, Schroder R, Kornblum C, Rudolph J, Wiesner RJ, Elger CE, Kunz WS.](http://www.ncbi.nlm.nih.gov/entrez/query.fcgi?db=pubmed&cmd=Retrieve&dopt=Citation&list_uids=15591266&query_hl=109&itool=pubmed_docsum) Tissue dependent co-segregation of the novel pathogenic G12276A mitochondrial tRNALeu(CUN) mutation with the A185G D-loop polymorphism. J Med Genet. 2004 Dec;41(12):e124. No abstract available. PMID: 15591266

74, [Zhadanov SI, Atamanov VV, Zhadanov NI, Oleinikov OV, Osipova LP, Schurr TG.](http://www.ncbi.nlm.nih.gov/entrez/query.fcgi?db=pubmed&cmd=Retrieve&dopt=Citation&list_uids=15922297&query_hl=107&itool=pubmed_docsum) A novel mtDNA ND6 gene mutation associated with LHON in a Caucasian family. Biochem Biophys Res Commun. 2005 Jul 15;332(4):1115-21. PMID: 15922297

75, [Rantamaki MT, Soini HK, Finnila SM, Majamaa K, Udd B.](http://www.ncbi.nlm.nih.gov/entrez/query.fcgi?db=pubmed&cmd=Retrieve&dopt=Citation&list_uids=16049925&query_hl=105&itool=pubmed_docsum) Adult-onset ataxia and polyneuropathy caused by mitochondrial 8993T-->C mutation. Ann Neurol. 2005 Aug;58(2):337-40. PMID: 16049925

76, [Esteitie N, Hinttala R, Wibom R, Nilsson H, Hance N, Naess K, Tear-Fahnehjelm K, von Dobeln U, Majamaa K, Larsson NG.](http://www.ncbi.nlm.nih.gov/entrez/query.fcgi?db=pubmed&cmd=Retrieve&dopt=Citation&list_uids=16044424&query_hl=104&itool=pubmed_docsum) Secondary metabolic effects in complex I deficiency. Ann Neurol. 2005 Oct;58(4):544-52. PMID: 16044424

77, [Kazuno AA, Munakata K, Mori K, Tanaka M, Nanko S, Kunugi H, Umekage T, Tochigi M, Kohda K, Sasaki T, Akiyama T, Washizuka S, Kato N, Kato T.](http://www.ncbi.nlm.nih.gov/entrez/query.fcgi?db=pubmed&cmd=Retrieve&dopt=Citation&list_uids=16048457&query_hl=101&itool=pubmed_docsum) Mitochondrial DNA sequence analysis of patients with 'atypical psychosis'. Psychiatry Clin Neurosci. 2005 Aug;59(4):497-503. PMID: 16048457

78, [Yuan H, Qian Y, Xu Y, Cao J, Bai L, Shen W, Ji F, Zhang X, Kang D, Mo JQ, Greinwald JH, Han D, Zhai S, Young WY, Guan MX.](http://www.ncbi.nlm.nih.gov/entrez/query.fcgi?db=pubmed&cmd=Retrieve&dopt=Citation&list_uids=16152638&query_hl=100&itool=pubmed_docsum) Cosegregation of the G7444A mutation in the mitochondrial COI/tRNA(Ser(UCN)) genes with the 12S rRNA A1555G mutation in a Chinese family with aminoglycoside-induced and nonsyndromic hearing loss. Am J Med Genet A. 2005 Oct 1;138(2):133-40. PMID: 16152638

79, [Merriwether DA, Hodgson JA, Friedlaender FR, Allaby R, Cerchio S, Koki G, Friedlaender JS.](http://www.ncbi.nlm.nih.gov/entrez/query.fcgi?db=pubmed&cmd=Retrieve&dopt=Citation&list_uids=16150714&query_hl=99&itool=pubmed_docsum) Ancient mitochondrial M haplogroups identified in the Southwest Pacific. Proc Natl Acad Sci U S A. 2005 Sep 13;102(37):13034-9. Epub 2005 Sep 6. Erratum in: Proc Natl Acad Sci U S A. 2005 Nov 15;102(46)16904. PMID: 16150714

80, [Kivisild T, Shen P, Wall DP, Do B, Sung R, Davis K, Passarino G, Underhill PA, Scharfe C, Torroni A, Scozzari R, Modiano D, Coppa A, de Knijff P, Feldman M, Cavalli-Sforza LL, Oefner PJ.](http://www.ncbi.nlm.nih.gov/entrez/query.fcgi?db=pubmed&cmd=Retrieve&dopt=Citation&list_uids=16172508&query_hl=95&itool=pubmed_docsum) The role of selection in the evolution of human mitochondrial genomes. Genetics. 2006 Jan;172(1):373-87. Epub 2005 Sep 19. PMID: 16172508

81, [Zhou X, Wei Q, Yang L, Tong Y, Zhao F, Lu C, Qian Y, Sun Y, Lu F, Qu J, Guan MX.](http://www.ncbi.nlm.nih.gov/entrez/query.fcgi?db=pubmed&cmd=Retrieve&dopt=Citation&list_uids=16364244&query_hl=90&itool=pubmed_DocSum) Leber's hereditary optic neuropathy is associated with the mitochondrial ND4 G11696A mutation in five Chinese families. Biochem Biophys Res Commun. 2006 Feb 3;340(1):69-75. Epub 2005 Dec 6. PMID: 16364244

82, [Behar DM, Metspalu E, Kivisild T, Achilli A, Hadid Y, Tzur S, Pereira L, Amorim A, Quintana-Murci L, Majamaa K, Herrnstadt C, Howell N, Balanovsky O, Kutuev I, Pshenichnov A, Gurwitz D, Bonne-Tamir B, Torroni A, Villems R, Skorecki K.](http://www.ncbi.nlm.nih.gov/entrez/query.fcgi?db=pubmed&cmd=Retrieve&dopt=Citation&list_uids=16404693&query_hl=89&itool=pubmed_docsum) The matrilineal ancestry of Ashkenazi Jewry: portrait of a recent founder event. Am J Hum Genet. 2006 Mar;78(3):487-97. Epub 2006 Jan 11. PMID: 16404693

83, [Zhadanov SI, Atamanov VV, Zhadanov NI, Schurr TG.](http://www.ncbi.nlm.nih.gov/entrez/query.fcgi?db=pubmed&cmd=Retrieve&dopt=Citation&list_uids=16418878&query_hl=87&itool=pubmed_docsum) De novo COX2 mutation in a LHON family of Caucasian origin: implication for the role of mtDNA polymorphism in human pathology. J Hum Genet. 2006;51(3):161-70. Epub 2006 Jan 18. PMID: 16418878

84, [Sun C, Kong QP, Palanichamy MG, Agrawal S, Bandelt HJ, Yao YG, Khan F, Zhu CL, Chaudhuri TK, Zhang YP.](http://www.ncbi.nlm.nih.gov/entrez/query.fcgi?db=pubmed&cmd=Retrieve&dopt=Citation&list_uids=16361303&query_hl=343&itool=pubmed_docsum) The dazzling array of basal branches in the mtDNA macrohaplogroup M from India as inferred from complete genomes. Mol Biol Evol. 2006 Mar;23(3):683-90. Epub 2005 Dec 16. PMID: 16361303

85, [Carelli V, Achilli A, Valentino ML, Rengo C, Semino O, Pala M, Olivieri A, Mattiazzi M, Pallotti F, Carrara F, Zeviani M, Leuzzi V, Carducci C, Valle G, Simionati B, Mendieta L, Salomao S, Belfort R Jr, Sadun AA, Torroni A.](http://www.ncbi.nlm.nih.gov/entrez/query.fcgi?db=pubmed&cmd=Retrieve&dopt=Citation&list_uids=16532388&query_hl=83&itool=pubmed_docsum) Haplogroup effects and recombination of mitochondrial DNA: novel clues from the analysis of Leber hereditary optic neuropathy pedigrees. Am J Hum Genet. 2006 Apr;78(4):564-74. Epub 2006 Jan 27. PMID: 16532388

86, [van Holst Pellekaan SM, Ingman M, Roberts-Thomson J, Harding RM.](http://www.ncbi.nlm.nih.gov/entrez/query.fcgi?db=pubmed&cmd=Retrieve&dopt=Citation&list_uids=16596590&query_hl=81&itool=pubmed_docsum) Mitochondrial genomics identifies major haplogroups in Aboriginal Australians. Am J Phys Anthropol. 2006 Oct;131(2):282-94. PMID: 16596590
