# Supplementary material for: Explaining the Imperfection of the Molecular Clock of Hominid Mitochondria
Source: PLoS One. 2009 Dec 29;4(12):e8260. doi: 10.1371/journal.pone.0008260 (PMC2794369; doi:10.1371/journal.pone.0008260)
Supplement: Table S1 — Mutation counts and coalescence ages of the 186 analyzed clades. (0.33 MB DOC) [file pone.0008260.s003.doc]

Table S1. Mutation counts and coalescence ages of the 186 analyzed clades

| Clade, motif ^a^ | N(ind) | *rho*S | age^b^ | SE(age) | *rho*AS | SE(*rho*AS) | N | S | RI |
| --- | --- | --- | --- | --- | --- | --- | --- | --- | --- |
| L0 1048-3516A-5442-9042-9347-10664-10915-13276 | 54 | 16.56 | 132300 | 17800 | 26.43 | 2.81 | 58 | 159 | 55 |
| L0-sub 1438-3756-4232-6815-8113A-8152-8251-12121-15466-15930-15941 | 19 | 8.47 | 67700 | 13800 | 15.32 | 2.47 | 22 | 54 | 16 |
| L0-sub 719-2706-3438-6266-13759 | 16 | 5.81 | 46400 | 11000 | 9.81 | 1.78 | 16 | 30 | 8 |
| L0a 5460-8428-8566-11176-12720-14308 | 28 | 5.75 | 45900 | 10400 | 9.75 | 1.78 | 20 | 50 | 20 |
| L0a-sub 5096 | 10 | 2.30 | 18400 | 5400 | 4.60 | 1.05 | 8 | 18 | 6 |
| L0a-sub 5147-5711-6257-8281d9bp-8460-11172 | 18 | 5.11 | 40800 | 10300 | 7.06 | 1.41 | 10 | 29 | 13 |
| L1-L2 4312-6185-10589-11914-12007 | 143 | 18.57 | 148400 | 18300 | 30.87 | 2.92 | 173 | 403 | 118 |
| L1 3666-7055-7389-13789-14178-14560 | 56 | 16.59 | 132500 | 18000 | 27.14 | 2.88 | 74 | 175 | 55 |
| L1c 2395d-5951-6071-8027-9072-10586-12810-13485-14000A-14911 | 32 | 12.88 | 102900 | 12700 | 20.66 | 2.09 | 54 | 132 | 44 |
| L1c-sub 6221A-6917-7055-11302-15226-15905-15978 | 6 | 6.33 | 50600 | 12600 | 13.67 | 2.24 | 15 | 23 | 13 |
| L1c-sub 12049-13149 | 9 | 10.89 | 87000 | 16500 | 17.67 | 2.57 | 17 | 44 | 15 |
| L1c-sub 3796T-3843-11899-14148 | 17 | 10.41 | 83200 | 14800 | 15.65 | 2.29 | 20 | 55 | 14 |
| L1b 709-1738-2352-3308-5036-5046-5655-6827-7867-8248-12519-14203-14769-15115 | 24 | 5.21 | 41600 | 15200 | 9.79 | 2.69 | 15 | 29 | 7 |
| L1b-sub 2768-5393 | 20 | 1.25 | 10000 | 3500 | 1.75 | 0.46 | 9 | 15 | 1 |
| L2 2416-8206-9221-10115-13590 | 87 | 11.33 | 90600 | 17400 | 17.46 | 2.42 | 88 | 192 | 52 |
| L2a 2789-7175-7274-7771-11914-13803-14566 | 59 | 5.29 | 42300 | 10700 | 7.46 | 1.41 | 41 | 100 | 19 |
| L2a-sub 12693-15784 | 51 | 3.00 | 24000 | 4500 | 4.90 | 0.70 | 36 | 79 | 16 |
| L2a-sub 3010-6663 | 10 | 2.50 | 20000 | 4900 | 4.20 | 0.88 | 6 | 19 | 5 |
| L2a-sub 3918-5285-15244-15629 | 13 | 1.85 | 14800 | 3800 | 2.38 | 0.54 | 5 | 18 | 1 |
| L2a-sub 5581 | 11 | 1.00 | 8000 | 2400 | 2.27 | 0.47 | 10 | 11 | 3 |
| L2b-L2c 1442-6724A-12236-15110-15217 | 23 | 5.26 | 42000 | 9700 | 14.09 | 2.21 | 34 | 50 | 18 |
| L2b 1706-2358-4158-4370-4767-5027-5331A-5814-6713-8080-8387-12948-14059 | 11 | 4.64 | 37000 | 9000 | 6.73 | 1.37 | 8 | 30 | 5 |
| L2b-sub 6026-10828-13924 | 7 | 2.43 | 19400 | 5500 | 3.57 | 0.82 | 3 | 14 | 4 |
| L2c-sub 13928C | 11 | 1.09 | 8700 | 2500 | 3.73 | 0.70 | 16 | 12 | 7 |
| L3e 2352-14212 | 30 | 4.80 | 38400 | 7100 | 7.47 | 1.05 | 18 | 60 | 26 |
| L3e1 14905 | 16 | 2.94 | 23500 | 5700 | 4.88 | 0.93 | 8 | 28 | 11 |
| L3e2 750 | 7 | 5.43 | 43400 | 10800 | 9.29 | 1.88 | 9 | 18 | 6 |
| L3e3 6221-6587-14152-15670-15942 | 7 | 2.14 | 17100 | 6800 | 3.29 | 0.94 | 1 | 9 | 7 |
| L3bd 13105 | 41 | 8.12 | 64900 | 11000 | 13.56 | 1.96 | 41 | 113 | 27 |
| L3d-sub 921 | 20 | 5.40 | 43100 | 7500 | 7.90 | 1.14 | 15 | 59 | 14 |
| L3b-sub 10373-15311-15944d | 16 | 3.00 | 24000 | 6200 | 4.75 | 0.87 | 16 | 25 | 8 |
| M* (xM2,G,Q,M7,M8,D) | 122 | 6.02 | 48100 | 3400 | 10.60 | 0.59 | 151 | 380 | 126 |
| M1 | 5 | 3.80 | 30400 | 8000 | 6.60 | 1.56 | 6 | 16 | 2 |
| M-1780-8502-11083-15670 | 5 | 6.80 | 54300 | 13000 | 10.20 | 1.97 | 7 | 22 | 4 |
| M5-709-3921-12477-14323 | 6 | 2.33 | 18600 | 6000 | 4.50 | 1.21 | 4 | 11 | 2 |
| M27 | 7 | 6.14 | 49100 | 11600 | 13.43 | 2.18 | 6 | 18 | 17 |
| M9 1041-3394-9242-14308 | 14 | 1.57 | 12600 | 3500 | 2.79 | 0.78 | 6 | 16 | 1 |
| M42 | 6 | 6.50 | 51900 | 12100 | 9.00 | 1.73 | 7 | 24 | 3 |
| M11 | 8 | 2.50 | 20000 | 4800 | 4.75 | 0.95 | 8 | 18 | 5 |
| M2 12007 | 28 | 2.57 | 20500 | 3000 | 6.18 | 0.73 | 46 | 55 | 30 |
| M2-sub 15431 | 14 | 1.50 | 12000 | 3100 | 4.07 | 0.61 | 18 | 17 | 15 |
| G 709-4833-5108 | 69 | 5.65 | 45200 | 11100 | 8.48 | 1.64 | 27 | 69 | 29 |
| G1 8200-15323-15497 | 28 | 3.36 | 26800 | 12000 | 5.14 | 1.73 | 8 | 21 | 11 |
| G1-sub 4793-11914-15860 | 23 | 0.65 | 5200 | 1500 | 1.43 | 0.32 | 3 | 13 | 8 |
| G2-sub 7600-9377-9575-14200 | 35 | 2.00 | 16000 | 4300 | 3.03 | 0.63 | 10 | 28 | 12 |
| Q 4117-5843-8790-12940 | 18 | 4.94 | 39500 | 8000 | 8.61 | 1.40 | 20 | 47 | 11 |
| M7 6455-9824 | 96 | 6.27 | 50100 | 11200 | 11.49 | 1.91 | 56 | 125 | 44 |
| M7a 2626-2772-4386-4958-12771 | 50 | 3.44 | 27500 | 9800 | 5.82 | 1.48 | 29 | 56 | 23 |
| M7a-sub 11017-11084 | 39 | 1.69 | 13500 | 2500 | 2.92 | 0.45 | 21 | 41 | 11 |
| M7bc 4071 | 46 | 6.17 | 49300 | 14700 | 11.22 | 2.54 | 27 | 66 | 18 |
| M7b-sub 10345 | 31 | 0.77 | 6200 | 1600 | 1.35 | 0.25 | 10 | 20 | 7 |
| M7c 4850-5442-11665-12091 | 9 | 3.22 | 25700 | 5900 | 5.00 | 0.96 | 6 | 24 | 7 |
| M8 4715-7196A-8584-15487T | 75 | 6.23 | 49800 | 9000 | 9.31 | 1.42 | 58 | 121 | 34 |
| M8-sub 6179-8684-14470 | 15 | 3.13 | 25000 | 6500 | 4.80 | 1.10 | 5 | 24 | 6 |
| Z 6752-9090-15784 | 21 | 3.29 | 26300 | 7300 | 5.57 | 1.19 | 17 | 28 | 9 |
| C 3552A-9545-11914-13263-14318 | 39 | 2.62 | 20900 | 3800 | 5.28 | 0.76 | 34 | 60 | 19 |
| C4 2232Ai-6026-11969-15204 | 12 | 3.17 | 25300 | 7500 | 4.25 | 1.03 | 4 | 16 | 5 |
| C-sub 15930 | 10 | 2.60 | 20800 | 4800 | 5.10 | 1.03 | 10 | 22 | 7 |
| D5 1107-5301-10397 | 38 | 4.61 | 36800 | 8600 | 8.97 | 1.59 | 26 | 64 | 21 |
| D5-sub 681-1048-5153-15724 | 12 | 3.58 | 28600 | 7600 | 5.75 | 1.34 | 6 | 25 | 7 |
| D5-sub 752-9180-11944-12026 | 23 | 2.09 | 16700 | 4600 | 4.43 | 1.09 | 12 | 26 | 7 |
| D4 3010-8414-14668 | 403 | 2.81 | 22400 | 3500 | 4.91 | 0.57 | 138 | 272 | 97 |
| D4-sub 2766 | 19 | 1.00 | 8000 | 4400 | 2.84 | 0.97 | 8 | 10 | 4 |
| D4-sub 13104 | 21 | 2.33 | 18600 | 7000 | 4.71 | 1.41 | 9 | 20 | 6 |
| D4-sub 11696 | 10 | 2.30 | 18400 | 3900 | 3.80 | 0.63 | 8 | 22 | 7 |
| D4-sub 3336-3644-5048 | 8 | 2.38 | 19000 | 6900 | 3.63 | 1.02 | 6 | 10 | 4 |
| D4a 3206-8473-14979 | 49 | 1.06 | 8500 | 1700 | 2.94 | 0.85 | 17 | 34 | 17 |
| D4a-sub10410 | 38 | 0.68 | 5500 | 1600 | 1.47 | 0.36 | 8 | 18 | 10 |
| D4-sub 1382C-8964-9824A | 63 | 2.89 | 23100 | 7400 | 4.03 | 1.01 | 21 | 51 | 14 |
| D4-sub 10104-15524 | 21 | 1.62 | 12900 | 4800 | 2.57 | 0.69 | 9 | 18 | 4 |
| D4-sub 9296 | 42 | 2.02 | 16200 | 6200 | 2.76 | 0.81 | 11 | 31 | 10 |
| D4-sub 14605 | 30 | 0.97 | 7700 | 2000 | 1.50 | 0.30 | 7 | 22 | 7 |
| D4b 10181-15440-15951 | 21 | 4.05 | 32300 | 9900 | 6.29 | 1.49 | 6 | 24 | 9 |
| D2 11215 | 70 | 2.80 | 22400 | 8700 | 5.34 | 1.52 | 18 | 30 | 6 |
| D2-sub 3316-9536 | 47 | 2.34 | 18700 | 9400 | 4.87 | 1.69 | 9 | 13 | 3 |
| D2-sub 15874 | 22 | 0.59 | 4700 | 1500 | 1.14 | 0.25 | 8 | 11 | 3 |
| D1 2092 | 19 | 2.16 | 17200 | 3600 | 3.79 | 0.61 | 15 | 27 | 9 |
| N*(xA,N1,N2,N9,R,X) | 18 | 4.61 | 36800 | 6800 | 10.22 | 1.07 | 47 | 53 | 23 |
| N* 8404 | 11 | 3.82 | 30500 | 6600 | 9.18 | 1.23 | 28 | 28 | 13 |
| N9 5417 | 84 | 3.27 | 26200 | 6900 | 5.55 | 1.10 | 43 | 90 | 23 |
| N9a 5231-12358-12372 | 42 | 1.88 | 15000 | 5300 | 3.57 | 0.93 | 21 | 35 | 18 |
| N9a-sub 961-15067 | 26 | 0.81 | 6500 | 2200 | 1.42 | 0.32 | 4 | 12 | 11 |
| N9-sub 5147-10607-11016-13183-14893 | 19 | 2.63 | 21000 | 5000 | 3.68 | 0.68 | 14 | 39 | 4 |
| N9-sub 12501-14893 | 10 | 2.00 | 16000 | 3800 | 2.80 | 0.58 | 7 | 18 | 0 |
| A 663-1736-4248-4824-8794 | 87 | 3.84 | 30700 | 7600 | 6.14 | 1.18 | 54 | 111 | 34 |
| A1 8563 | 47 | 4.79 | 38300 | 13300 | 5.87 | 1.74 | 10 | 41 | 14 |
| A1-sub 4655-11647 | 40 | 3.10 | 24800 | 10700 | 4.10 | 1.45 | 8 | 29 | 10 |
| A1-sub 5773-12880 | 22 | 0.59 | 4700 | 1500 | 0.91 | 0.22 | 4 | 11 | 3 |
| A2 8027 | 29 | 2.90 | 23100 | 6900 | 4.59 | 0.92 | 28 | 48 | 11 |
| A2-sub 12007 | 23 | 2.04 | 16300 | 3100 | 3.61 | 0.52 | 21 | 36 | 8 |
| W 709-1243-3505-5046-5460-8994-11674-11947-12414-15884C | 55 | 3.31 | 26400 | 10000 | 4.27 | 1.29 | 15 | 39 | 10 |
| W3 1406-13263-15784 | 8 | 1.75 | 14000 | 3700 | 2.38 | 0.54 | 4 | 14 | 1 |
| W1 7864 | 42 | 2.36 | 18800 | 10000 | 3.19 | 1.31 | 11 | 14 | 3 |
| W1a 5495-12669 | 36 | 0.50 | 4000 | 2900 | 1.33 | 0.58 | 7 | 6 | 2 |
| X 1719-6221-6371-13966-14470 | 28 | 1.96 | 15700 | 2400 | 4.32 | 0.77 | 19 | 48 | 8 |
| N1 1719-8251-10238-12501 | 43 | 5.49 | 43900 | 9300 | 12.21 | 2.32 | 48 | 70 | 23 |
| I 4529T-10034-15924 | 36 | 3.47 | 27700 | 7200 | 5.86 | 1.09 | 32 | 48 | 13 |
| U 11467-12308-12372 | 363 | 6.74 | 53900 | 8100 | 12.08 | 1.47 | 259 | 527 | 163 |
| U2-sub 13734 | 20 | 2.20 | 17600 | 3000 | 4.65 | 0.49 | 15 | 16 | 11 |
| U3 14139-15454 | 13 | 5.08 | 40600 | 11000 | 9.38 | 1.93 | 11 | 31 | 11 |
| U7 980-5360 | 10 | 5.20 | 41500 | 11100 | 8.20 | 1.74 | 12 | 23 | 1 |
| U4/U9 5999 | 20 | 6.85 | 54700 | 14200 | 9.45 | 2.02 | 15 | 37 | 10 |
| U8 9698 | 148 | 6.98 | 55800 | 15600 | 12.29 | 2.60 | 87 | 175 | 44 |
| K 3480-10550-11299-14798 | 140 | 2.94 | 23500 | 3600 | 6.39 | 1.23 | 76 | 153 | 43 |
| K2 9716 | 32 | 2.06 | 16500 | 3300 | 4.41 | 1.26 | 11 | 33 | 6 |
| K2-sub 709-4561 | 26 | 1.65 | 13200 | 3400 | 2.04 | 0.44 | 6 | 22 | 4 |
| K1 1189-10398 | 108 | 2.91 | 23200 | 3900 | 4.69 | 0.58 | 64 | 119 | 36 |
| K1-sub 11914 | 28 | 2.82 | 22500 | 8800 | 4.68 | 1.41 | 13 | 22 | 9 |
| K1-sub 11485 | 10 | 2.60 | 20800 | 5400 | 3.90 | 0.83 | 5 | 18 | 4 |
| K1-sub 9093-11377 | 13 | 0.38 | 3100 | 1400 | 1.15 | 0.41 | 3 | 5 | 3 |
| K1-sub 5913 | 11 | 3.82 | 30500 | 7900 | 6.64 | 1.35 | 12 | 21 | 6 |
| K1-sub 6260-11840-13740 | 10 | 2.00 | 16000 | 7600 | 3.70 | 1.15 | 10 | 12 | 1 |
| U1-sub 2218-4991-6062-7581-14364 | 9 | 3.89 | 31100 | 9300 | 6.11 | 1.26 | 15 | 20 | 5 |
| U6 3348 | 16 | 3.56 | 28500 | 6600 | 6.50 | 1.15 | 16 | 33 | 9 |
| U6-sub 7805-14179 | 9 | 4.22 | 33700 | 7900 | 6.11 | 1.15 | 7 | 24 | 6 |
| U5 3197-9477-13617 | 113 | 3.41 | 27200 | 7600 | 6.35 | 1.18 | 65 | 123 | 46 |
| U5a1 14793 | 46 | 2.04 | 16300 | 2600 | 4.39 | 0.82 | 31 | 59 | 13 |
| U5a1-sub 15218 | 28 | 1.68 | 13400 | 2800 | 3.86 | 0.77 | 19 | 32 | 11 |
| U5a1-sub 9667 | 15 | 1.00 | 8000 | 2700 | 1.87 | 0.43 | 8 | 10 | 4 |
| U5ab 7768-14182 | 67 | 2.34 | 18700 | 5900 | 5.00 | 1.09 | 33 | 62 | 33 |
| U5a 1721-13637 | 18 | 1.94 | 15500 | 4100 | 5.39 | 1.14 | 15 | 25 | 15 |
| U5a-sub 4732 | 15 | 1.60 | 12800 | 4600 | 3.87 | 0.86 | 8 | 15 | 10 |
| U5b 5656 | 43 | 2.79 | 22300 | 9000 | 3.56 | 1.14 | 14 | 36 | 15 |
| U5b-sub 12618 | 36 | 1.86 | 14900 | 7100 | 2.64 | 0.90 | 11 | 22 | 14 |
| U5b-sub 7385 | 19 | 0.47 | 3800 | 1300 | 1.32 | 0.28 | 7 | 9 | 7 |
| J 10398-12612-13708 | 167 | 2.17 | 17400 | 2300 | 6.15 | 1.18 | 96 | 171 | 62 |
| J2 7476-15257 | 29 | 4.24 | 33900 | 8300 | 6.41 | 1.35 | 14 | 48 | 10 |
| J2b 5633-10172-15812 | 17 | 2.12 | 16900 | 3700 | 2.88 | 0.52 | 6 | 27 | 5 |
| J2a 10499-11377 | 12 | 3.83 | 30600 | 11000 | 5.17 | 1.47 | 7 | 18 | 4 |
| J1 3010 | 138 | 1.74 | 13900 | 2200 | 4.67 | 0.93 | 81 | 123 | 50 |
| J1b 8269 | 20 | 1.90 | 15200 | 8000 | 6.55 | 2.10 | 13 | 13 | 10 |
| J1b-sub 8557 | 17 | 0.82 | 6600 | 4000 | 1.71 | 0.55 | 7 | 6 | 7 |
| J1c 14798 | 112 | 1.44 | 11500 | 1700 | 3.12 | 0.37 | 62 | 97 | 35 |
| J1c-sub 13934 | 18 | 1.72 | 13800 | 3600 | 3.00 | 0.54 | 10 | 21 | 9 |
| J1c-sub 3394 | 20 | 1.30 | 10400 | 3400 | 1.90 | 0.54 | 3 | 11 | 5 |
| J1c-sub 5198 | 15 | 1.47 | 11700 | 2800 | 3.07 | 0.63 | 7 | 20 | 7 |
| T 709-1888-4917-8697-10463-13368-14905-15607-15928 | 140 | 3.87 | 30900 | 8800 | 5.43 | 1.22 | 63 | 147 | 38 |
| T1 12633A | 41 | 1.66 | 13300 | 6200 | 2.24 | 0.79 | 13 | 31 | 6 |
| T1-sub 9899 | 31 | 0.90 | 7200 | 1700 | 1.39 | 0.27 | 7 | 22 | 5 |
| T2-sub 11812 | 98 | 2.41 | 19200 | 4800 | 4.36 | 0.85 | 49 | 113 | 30 |
| T2-sub 13965-14687 | 15 | 2.27 | 18100 | 6300 | 4.27 | 1.11 | 10 | 17 | 5 |
| T2-sub 930-5147 | 53 | 1.25 | 9900 | 1400 | 2.08 | 0.27 | 25 | 49 | 9 |
| HV(xH,V) 14766 | 27 | 2.33 | 18600 | 2600 | 3.81 | 0.48 | 22 | 51 | 12 |
| V 4580 | 71 | 0.90 | 7200 | 1500 | 2.49 | 0.48 | 26 | 39 | 12 |
| H 2706-7028 | 553 | 1.86 | 14800 | 1500 | 3.68 | 0.42 | 258 | 484 | 188 |
| H1 3010 | 187 | 1.13 | 9100 | 1300 | 2.12 | 0.20 | 81 | 141 | 52 |
| H1c1 9150 | 18 | 0.28 | 2200 | 1200 | 0.61 | 0.24 | 2 | 4 | 2 |
| H1b 3796 | 9 | 0.78 | 6200 | 2200 | 1.67 | 0.43 | 2 | 7 | 6 |
| H1a1 6365 | 15 | 0.40 | 3200 | 1300 | 0.93 | 0.27 | 4 | 6 | 3 |
| H4-sub 9123-14365-14582 | 25 | 1.08 | 8600 | 2500 | 2.88 | 0.73 | 13 | 17 | 12 |
| H5 4336 | 32 | 1.47 | 11700 | 5900 | 2.44 | 0.77 | 16 | 23 | 8 |
| H5-sub 4336-15833 | 23 | 0.74 | 5900 | 1700 | 1.48 | 0.31 | 8 | 15 | 5 |
| H6a 4727-9380 | 11 | 1.55 | 12300 | 3500 | 3.18 | 0.90 | 7 | 15 | 4 |
| H6a-sub 11253 | 8 | 1.63 | 13000 | 3600 | 2.75 | 0.59 | 6 | 13 | 3 |
| H2 1438 | 34 | 2.09 | 16700 | 7500 | 4.32 | 1.13 | 14 | 32 | 18 |
| H2-sub 4769 | 31 | 1.10 | 8800 | 2000 | 3.23 | 0.73 | 11 | 25 | 11 |
| H2b 750 | 15 | 0.80 | 6400 | 2100 | 2.80 | 0.86 | 7 | 10 | 5 |
| H2b-sub 8860 | 8 | 0.88 | 7000 | 2600 | 1.63 | 0.45 | 3 | 7 | 3 |
| H3 6776 | 61 | 1.07 | 8500 | 1400 | 2.25 | 0.28 | 29 | 47 | 24 |
| H10 14470A | 11 | 0.64 | 5100 | 2200 | 2.27 | 0.54 | 12 | 6 | 3 |
| H13 14872 | 18 | 3.89 | 31100 | 11500 | 6.50 | 1.78 | 5 | 18 | 12 |
| H13-sub 4745-13680 | 15 | 2.47 | 19700 | 7800 | 4.13 | 1.17 | 4 | 13 | 6 |
| H13-sub 7337-13326 | 9 | 1.22 | 9800 | 5800 | 4.00 | 1.29 | 4 | 4 | 6 |
| H11-sub 961G | 7 | 1.29 | 10300 | 4400 | 1.86 | 0.62 | 1 | 7 | 3 |
| H7 4793 | 19 | 1.32 | 10500 | 3400 | 2.37 | 0.53 | 10 | 18 | 7 |
| H7-sub 5348 | 7 | 1.29 | 10300 | 3200 | 1.57 | 0.47 | 1 | 9 | 1 |
| F 6392-10310 | 57 | 6.46 | 51600 | 12000 | 11.63 | 2.04 | 43 | 96 | 37 |
| F1 6962-10609-12406-12882 | 46 | 4.50 | 36000 | 9300 | 7.78 | 1.50 | 25 | 60 | 25 |
| R* (xB,HV,JT,R9,U,15607) | 34 | 7.41 | 59200 | 6200 | 12.97 | 1.00 | 75 | 164 | 50 |
| R5 | 10 | 5.10 | 40700 | 12200 | 7.10 | 1.68 | 8 | 22 | 5 |
| R30 8584 | 5 | 9.40 | 75100 | 13100 | 16.00 | 2.38 | 13 | 31 | 8 |
| R31 15884 | 4 | 9.75 | 77900 | 15100 | 15.50 | 2.37 | 12 | 30 | 6 |
| R* 15607 (incl P) | 20 | 7.35 | 58700 | 6600 | 12.25 | 1.05 | 48 | 111 | 28 |
| P 6077-10118 | 6 | 5.00 | 40000 | 8100 | 8.33 | 1.35 | 13 | 26 | 4 |
| B 8281del9bp | 162 | 6.55 | 52300 | 7300 | 11.56 | 1.20 | 137 | 263 | 80 |
| B4a 5465-9123 | 40 | 4.05 | 32400 | 9300 | 6.55 | 1.38 | 26 | 57 | 20 |
| B4a-sub 709-10238 | 14 | 2.79 | 22300 | 5400 | 4.64 | 0.82 | 11 | 23 | 9 |
| B4bd 827-15535 | 51 | 6.37 | 50900 | 13000 | 9.63 | 1.79 | 57 | 88 | 19 |
| B4b 4820-13590 | 45 | 4.76 | 38000 | 9400 | 7.80 | 1.44 | 40 | 69 | 14 |
| B4b-sub 3547-4977-6473-9950-11177 | 20 | 2.45 | 19600 | 3100 | 4.05 | 0.50 | 25 | 43 | 4 |
| B-sub 709-1119-3497-15346 | 19 | 3.42 | 27300 | 7100 | 5.42 | 1.04 | 14 | 31 | 9 |
| B5 709-8584-9950-10398 | 40 | 6.80 | 54300 | 12800 | 12.43 | 2.36 | 22 | 60 | 20 |
| B5-sub 3537-15235 | 10 | 4.10 | 32800 | 10100 | 5.60 | 1.46 | 2 | 15 | 4 |
| B5b 1598-8829-12361-15223-15508-15662-15851-15927 | 30 | 4.03 | 32200 | 8700 | 6.03 | 1.20 | 17 | 40 | 14 |
| B5b-sub 4895-15850 | 16 | 1.88 | 15000 | 4400 | 4.31 | 0.93 | 10 | 21 | 8 |
| B5b-sub 11146-14470 | 11 | 1.64 | 13100 | 6300 | 2.82 | 1.09 | 1 | 10 | 5 |
|  |  |  |  |  |  |  |  |  |  |
| ^a^ Clade names and defining mutation motifs of the clades are as in Figure S1 |  |  |  |  |  |  |  |  |  |
| ^b^ Coalescence ages are according to the rate of 1 synonymous substitution per 7990 years | |  |  |  |  |  |  |  |  |
| N-non-synonymous |  |  |  |  |  |  |  |  |  |
| S-synonymous |  |  |  |  |  |  |  |  |  |
| ST-synonymous transition |  |  |  |  |  |  |  |  |  |
| RI-RNA and intergenic |  |  |  |  |  |  |  |  |  |
