# Supplementary material for: Explaining the Imperfection of the Molecular Clock of Hominid Mitochondria
Source: PLoS One. 2009 Dec 29;4(12):e8260. doi: 10.1371/journal.pone.0008260 (PMC2794369; doi:10.1371/journal.pone.0008260)
Supplement: Table S2 — Corrected misannotations of the mutations on the Mitomap tree. (0.05 MB DOC) [file pone.0008260.s004.doc]

Table S2. Corrected misannotations of the mutations on the phylogenetic tree of mtDNA coding sequences from www.mitomap.org

|  | **Mitomap** |  |  | **corrected** |  |
| --- | --- | --- | --- | --- | --- |
| nucleotide | color code | designation | # a | corrected | gene |
| 7988 | synonymous | COII | 1 | L135F | COII |
| 8269 | noncoding | " " | 5 | synonymous | COII |
| 8396 | synonymous | ATP8 | 1 | T11A | ATP8 |
| 8911 | nonsynonymous | H109RATP6 | 1 | synonymous | ATP6 |
| 9196 | synonymous | ATP6 | 1 | D224N | ATP6 |
| 9375 | synonymous | COIII | 1 | W57R | COIII |
| 9966 | synonymous | COIII | 1 | V254I | COIII |
| 11065 | nonsynonymous | F3LND4 | 1 | synonymous | ND4 |
| 13149 | nonsynonymous | D224NND5 | 1 | synonymous | ND5 |
| 13594 | synonymous | ND5 | 1 | S420G | ND5 |
| 13741 | synonymous | ND5 | 1 | T469A | ND5 |
| 13879 | synonymous | ND5 | 1 | S515P | ND5 |
| 14148 | noncoding | " " | 3 | synonymous | ND5 |
| 15589 | RNA | T | 1 | synonymous | cytb |

^a^ Number of misannotations on the tree. In most cases, only a minority of re-occurrences of a particular mutation were misannotated with most of the annotations being correct.
