# Supplementary material for: Explaining the Imperfection of the Molecular Clock of Hominid Mitochondria
Source: PLoS One. 2009 Dec 29;4(12):e8260. doi: 10.1371/journal.pone.0008260 (PMC2794369; doi:10.1371/journal.pone.0008260)
Supplement: Figure S1 — Mitomap tree. Phylogenetic tree of human mitochondrial DNA coding region (positions 577-16023) sequences from Mitomap web page as of March, 2007 (www.mitomap.org). Inside the figure the legend explains the color code for the substitution classes. Identified mis-assignments (Table S2) have been underlined and the corrections maintaining the original color scheme have been added to the figure. (0.50 MB PDF) [file pone.0008260.s002.pdf]

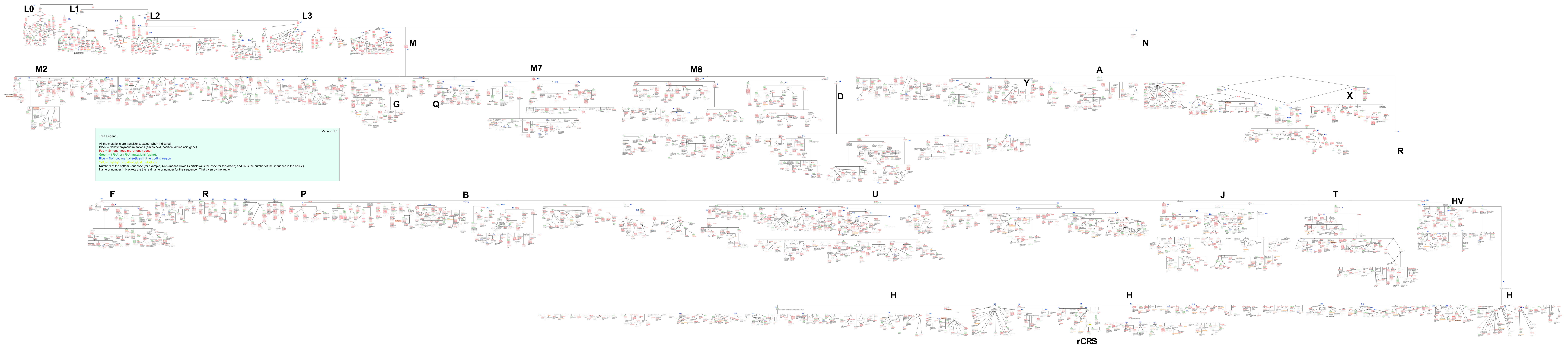

L0
L1
L2
L3
M
N
M2
M7
M8
Y
A
X
G
Q
D
R
Version 1.1
Tree Legend:
All the mutations are transitions, except when indicated.
Black = Nonsynonymous mutations (amino acid, position, amino acid,gene)
Red = Synonymous mutations (gene)
Green = tRNA or rRNA mutations (gene).
Blue = Non coding nucleotides in the coding region
Yellow highlight = pathological mutations
Numbers at the bottom - our code (for example, 4(55) means Howell's article (4 is the code for this article) and 55 is the number of the sequence in the article).
Name or number in brackets are the real name or number for the sequence. That given by the author.
F
R
P
B
U
J
T
HV
H
H
H
rCRS
